# Geotechnical characterization and stability analysis of subaqueous slopes in Lake Lucerne (Switzerland)

**DOI:** 10.1007/s11069-022-05310-1

**Published:** 2022-03-29

**Authors:** Anastasiia Shynkarenko, Katrina Kremer, Sylvia Stegmann, Paolo Bergamo, Agostiny Marrios Lontsi, Alexander Roesner, Steffen Hammerschmidt, Achim Kopf, Donat Fäh

**Affiliations:** 1 Swiss Seismological Service (SED), ETH Zürich, Sonneggstrasse 5, 8092 Zurich, Switzerland; 2 Geological Institute and Oeschger Centre for Climate Change Research, University of Bern, Baltzerstrasse 1+3, 3012 Bern, Switzerland; 3 MARUM—Center for Marine Environmental Sciences, University of Bremen, Leobener Strasse 8, 28359 Bremen, Germany

**Keywords:** Lake Lucerne, Slope stability, Subaqueous landslides, Cone penetration testing, Sediment coring, Undrained shear strength

## Abstract

Tsunamis occur not only in marine settings but also in lacustrine environments. Most of the lacustrine tsunamis are caused by seismically- or aseismically-triggered mass movements. Therefore, an assessment of the stability of subaqueous slopes is crucial for tsunami hazard assessment in a lake. We selected Lake Lucerne (Switzerland) as a natural laboratory to perform an in-depth geotechnical characterization of its subaqueous slopes. This lake experienced documented tsunamis in 1601 and 1687. Some of its slopes still bear sediment volumes with a potential for tsunamigenic failure. To identify such slopes, we interpreted available reflection seismic data and analyzed the bathymetric map. Then, we performed 152 dynamic Cone Penetration Tests with pore pressure measurement (CPTu) and retrieved 49 sediment cores at different locations in the lake. These data were used to characterize the failure-prone sediments and to evaluate the present-day static stability of subaqueous slopes. Obtained results allowed the definition of three classes of slopes in terms of static stability: unstable slopes, stable slopes close to the unstable state, and stable areas. Non-deltaic slopes with thicker unconsolidated fine-grained sediment drape and moderate-to-high slope gradients (> 5–10°) have the lowest Factor of Safety. In agreement with previous studies, the failure plane for the non-deltaic slopes is embedded within the fine-grained glaciolacustrine sediments. Deltaic slopes with prevailing coarse-grained sediments mostly appear statically stable. Finally, we generalized the measured undrained shear strength profiles ${s}_{u}(z)$ into the depth-dependent power-law models. These models define the ${s}_{u}$ of Lake Lucerne’s sediments and can be applied to other lakes with similar sedimentation history.

**Supplementary Information:**

The online version contains supplementary material available at 10.1007/s11069-022-05310-1.

## Introduction

On very rare occasions, tsunamis can occur in lakes as a consequence of subaqueous mass movements (Schnellmann et al. 2002, 2004; Gisler et al. 2004; Moernaut et al. 2007; Schwarz-Zanetti et al. 2018; Sammartini et al. 2019; Kremer et al. 2020). These mass movements can be caused by seismic events (e.g., an earthquake) or specific aseismic phenomena (e.g., sediment overloading or accumulation of excess pore water pressure). Due to the growth in population and infrastructure along the lake shores, an assessment of the mass movement-induced lake tsunami hazard is required.

For a proper study of the tsunami hazard, the stability of the lake slopes has to be assessed. In this work, we focus on the static stability of subaqueous slopes. Among the most widely used approaches to evaluate the static slope stability are the limit equilibrium method (LEM; Morgenstern and Price 1965; Kramer 1996), the infinite slope method (1D LEM; Morgenstern 1967), the finite element (FEM) and finite difference (FDM) methods (Mansour and Kalantari 2011; Baba et al. 2012; Chatzi and Escallon 2013). FEM or FDM allow a comprehensive analysis of the target structure (De Martin 2010; Smith et al. 2016; Stoecklin et al. 2017; Carlton et al. 2019) but require also detailed site information, uncertainty estimates and understanding of the nonlinear behavior of sediments under seismic loading. Consequently, they have high computational demands and are rarely used to analyze the stability of slopes on large areas. In contrast, the infinite slope and LEM methods are quite conservative and simplistic but allow a rapid assessment of the slope stability at multiple locations and large areas, thus they are widely used in different case studies (Strasser et al. 2011; Duncan et al. 2014; Strupler et al. 2018a; Carlton et al. 2019). The most crucial parameters for the stability analysis are the undrained shear strength and the unit weight of the sediments together with the slope angle (e.g., Sultan et al. 2010; Ai et al. 2014; Stegmann and Kopf 2014; Wiemer et al. 2015; Strupler et al. 2020). To derive these parameters, detailed morphological and geotechnical (in situ and/or laboratory) site investigations are required.

Swiss lakes have experienced tsunamis on historical and prehistorical timescales (Schwarz-Zanetti et al. 2003; Schnellmann et al. 2004; Kremer et al. 2012; Hilbe and Anselmetti 2014a; Nigg et al. 2021) and represent ideal “field laboratories” for subaqueous slope stability investigations. We selected Lake Lucerne in Central Switzerland as a case study site as this lake has experienced both seismically- and aseismically-triggered mass movement-tsunamis in the past (e.g., in 1601 AD and 1687 AD; Siegenthaler et al. 1987; Strasser et al. 2007, 2011; Hilbe et al. 2011) and a large dataset obtained by past investigations is already available for it. Notwithstanding the past failures, Lake Lucerne still bears a wide range of sediment-loaded slopes with an unknown failure potential and, thus, a necessity for a stability assessment.

Within the past investigations in Lake Lucerne, a multitude of geological, geotechnical and geophysical data has been collected, in particular, a high-resolution bathymetry map of the lake floor, a dense grid of reflection seismic profiles, and seismic ambient vibration measurements; also, the chronology of past mass movements has been established (Finckh et al. 1984; Schnellmann et al. 2002, 2004, 2006; Stegmann et al. 2007; Strupler 2012; Hilbe and Anselmetti 2014b; Hammerschmidt 2019; Shynkarenko et al. 2021; Lontsi et al. 2022). The question of the present-day and past stability of subaqueous slopes in Lake Lucerne was addressed using the LEM in Strasser et al. (2007, 2011), Hilbe and Anselmetti (2015) and Strupler et al. (2020). These studies showed that the failure plane for the lateral non-deltaic slopes is embedded within the glaciolacustrine sediments, thus the failure-prone sediment drape consists of the fine-grained lacustrine and glaciolacustrine sediments and usually has a thickness between 0 and 15 m depending on the slope morphology and history of the past mass movements. However, these investigations generally covered three out of seven sub-basins of the lake (except Hilbe and Anselmetti 2015 and Strupler et al. 2020, who considered the entire lake excluding deltaic areas). Moreover, the input parameters to the stability analysis were derived from the limited geotechnical data obtained at a few isolated locations on the lateral hemipelagic slopes. Beyond that, no tests have been previously performed at the deltaic slopes. Consequently, there is only limited information on the spatial variability of sediment properties. The possibility to extrapolate such data over large areas and the applicability of the geotechnical measurements done in Lake Lucerne to other lakes should be verified by a denser grid of measurements. Additionally, to address the stability state of deltaic slopes, the geotechnical testing of sediment cover at these areas is required.

To address the open questions, we perform extensive in situ CPTu investigations, sediment coring and laboratory measurements on diverse slopes and slope-perpendicular transects within Lake Lucerne. In combination with the previously acquired datasets, this study aims at (1) high-resolution investigation of the geotechnical properties of Lake Lucerne sediments, (2) assessing the stability of selected sediment-loaded subaqueous slopes using the infinite slope method, and (3) proposing the generalized models of the undrained shear strength for the failure-prone lake sediments. These models can be transferred to other Swiss lakes with a similar sedimentation history. Therefore, this study will constitute an important input for the lake tsunami hazard assessment. Moreover, considering lakes as models for ocean or sea margins but with easier access to the measurement sites and smaller spatial extent (Strasser et al. 2007; Strupler et al. 2017), the approach used for the lacustrine slopes and acquired knowledge may be transferred to marine settings.

## Geological setting, previous studies and site selection

### Geological setting

**Fig. 1:**
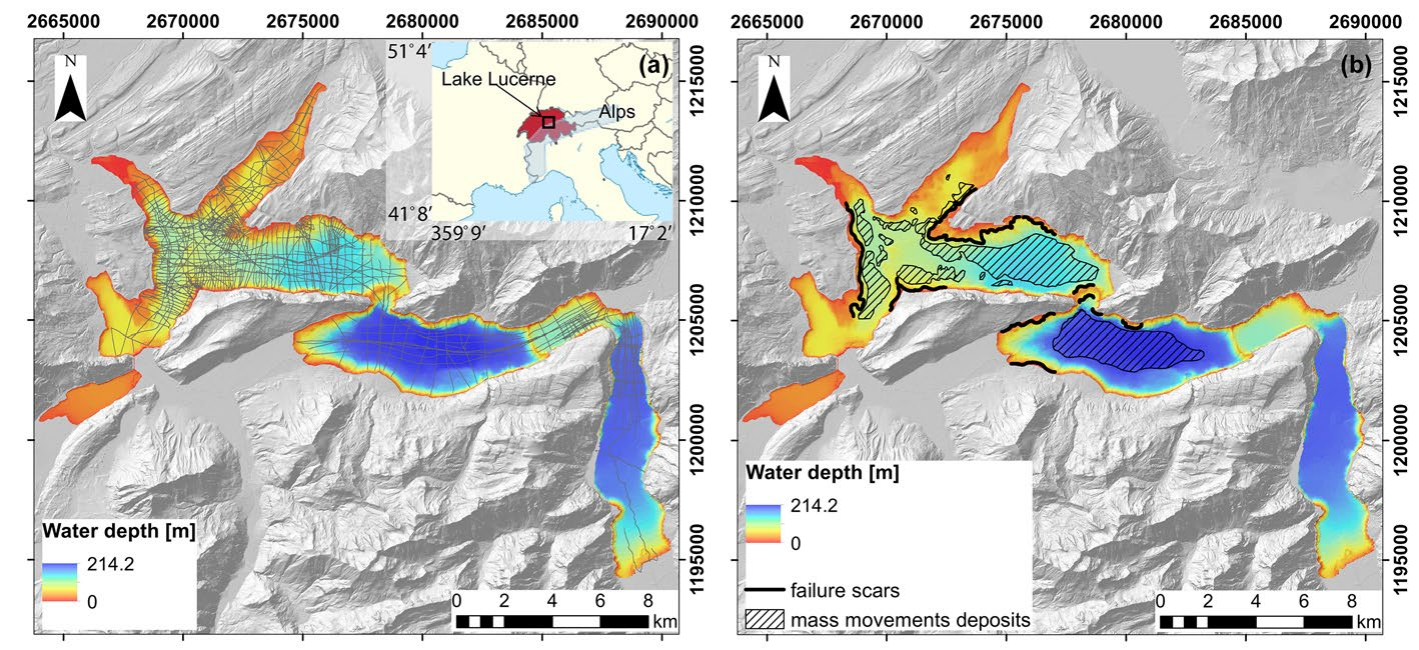
**a** Bathymetry map of Lake Lucerne (Source: Federal Office of Topography swisstopo; Hilbe et al. 2011; Hilbe and Anselmetti 2014a); available reflection seismic profiles are marked with gray lines. The inset is given in WGS84 coordinates and shows the location of Lake Lucerne. **b** Schematic representation of the failure scars and mass movement deposits triggered by the *M*_w_ 5.9 Unterwalden earthquake in 1601 (modified after Schnellmann et al. 2002, Strasser et al. 2011 and Hilbe and Anselmetti 2014a). The coordinates are given in the Swiss Coordinate System LV95 with units of m

Lake Lucerne is a fjord-type perialpine lake in Central Switzerland (Fig. 1). It covers an area of 114 km^2^ and has 7 sub-basins, some of which are separated by underwater moraine ridges. The maximum depth of the lake is 214 m. The bedrock beneath Lake Lucerne is represented by Helvetic nappes (consisting of Mesozoic marine limestone, marls and shales) in the Alpine region (south-eastern part of the lake) and by Swiss Molasse (Tertiary clastic sedimentary rocks) in the Foreland Basin (north-western part of the lake). The main rivers that enter the lake are Reuss, Muota, Engelberger Aa and Sarner Aa, while the main outflow is Reuss river. The maximum thickness of unconsolidated (glacial to postglacial) lake sediments is about 200 m in the deep basins and up to ca. 15 m on the lateral (non-deltaic) slopes (Finckh et al. 1984; Pfiffner et al. 1990, 2002; Greber et al. 1994; Strasser et al. 2007; Hilbe et al. 2011; Hilbe and Anselmetti 2014a, 2014b; Pfiffner 2018).

The main lithological units deposited on the subaqueous non-deltaic slopes (i.e., lateral slopes and slopes on moraine ridges) of Lake Lucerne are defined as follows (from top to bottom): (1) Holocene fine-grained lacustrine sediments, (2) fine-grained glaciolacustrine sediments of the Late Glacial period and (3) glacial sediments (e.g., till; Strasser et al. 2007; Strupler 2012; Hilbe and Anselmetti 2014b; Sammartini et al. 2021). In this study, for the sake of simplicity, we will use the following terminology for these lithological units: lacustrine, glaciolacustrine, and glacial sediments, respectively. On the lateral non-deltaic slopes (in following, we will only use the term “lateral slopes” to simplify) and moraine ridge slopes, traces of the mass movements are revealed on the digital bathymetric map and reflection seismic data (e.g., Schnellmann et al. 2005; Strasser et al. 2007; Hilbe and Anselmetti 2014b). The failure plane is usually embedded within the glaciolacustrine sediments (e.g., Stegmann et al., 2007; Strasser et al. 2011). In contrast, the sediments on deltaic slopes typically consist of an interchanging sequence of fine- to coarse-grained Holocene sediments with variable and mixed grain size distributions. In this study, we will use the term “deltaic sediments” to refer to this lithological unit. Although mass movements have been also described on deltaic slopes in previous studies (Hilbe and Anselmetti 2014a; Sammartini et al. 2019), no clear location of the failure plane has been found for them.

The most probable triggers of the slope failures in Swiss lakes are earthquake shaking or sediment overloading/excess pore water pressure (Stegmann et al. 2007; Wiemer and Kopf 2017; Roesner et al. 2019; Kremer et al. 2020). Although Switzerland is a country with moderate seismicity where events with a moment magnitude *M*_w_ ≥ 5.5 occur quite rarely (28 of such events have been identified for the past 700 years; Fäh et al. 2016; Wiemer et al. 2016; Cauzzi et al. 2018), there are historical reports and prehistorical traces of earthquake-triggered tsunamis in Swiss lakes and Lake Lucerne in particular.

### Previously acquired datasets

During previous studies in Lake Lucerne, a multitude of geomorphic, sedimentological and geophysical data which contribute to the present study have been acquired, in particular:

1. A high-resolution (1 m × 1 m) bathymetry map of the lake floor (Fig. 1a and b), acquired between 2007 and 2012 using a GeoAcoustics GeoSwath Plus interferometric sonar and Kongsberg EM2040 multibeam echosounder (Hilbe et al. 2011; Hilbe and Anselmetti 2014b). This map contributes to the morphometric analysis of the lake floor and allows us to detect past failures and to derive the slope angle map;
2. A grid of reflection seismic profiles (Fig. 1a) obtained using three types of seismic sources: 1 in^3^ (ca. 16.4 cm^3^) airgun with 120–1600 Hz central frequency; single-channel 3.5 kHz pinger source; and “centipede-sparker” with 150–1500 Hz central frequency (Schnellmann et al. 2002, 2005, 2006; Hilbe et al. 2011; Strupler 2012; Sammartini et al. 2021). These data allow the identification of lithological units deposited on different slopes and the derivation of sediments’ thickness maps;
3. History of previous mass-movements, back-analysis of slope stability and failed sediment volumes, locations of the failure scars (e.g., Fig. 1b), and possible triggering events (Schnellmann et al. 2002, 2005; Stegmann et al. 2007; Hilbe et al. 2011; Strasser et al. 2011; Hilbe and Anselmetti 2014a, 2014b; Hammerschmidt 2019).

### Site selection

Based on the available datasets described in Sect. 2.2, we defined the following criteria to select the investigation sites: (1) thickness of available sediment packages derived from the reflection seismic profiles; (2) slope gradient derived from the bathymetry map of the lake floor; (3) history of the previous mass-movements in the lake. More specifically, our target sites correspond to the areas which have not failed recently, bear potentially tsunamigenic sediment volumes (Strupler et al. 2020) and have a slope gradient lower than 20–25° (the majority of landslides occurs on slopes > 5–10°; slopes steeper than 20–25° usually do not have considerable sediment accumulation; see Hilbe and Anselmetti 2015; Strupler et al. 2017, 2018a).

**Fig. 2:**
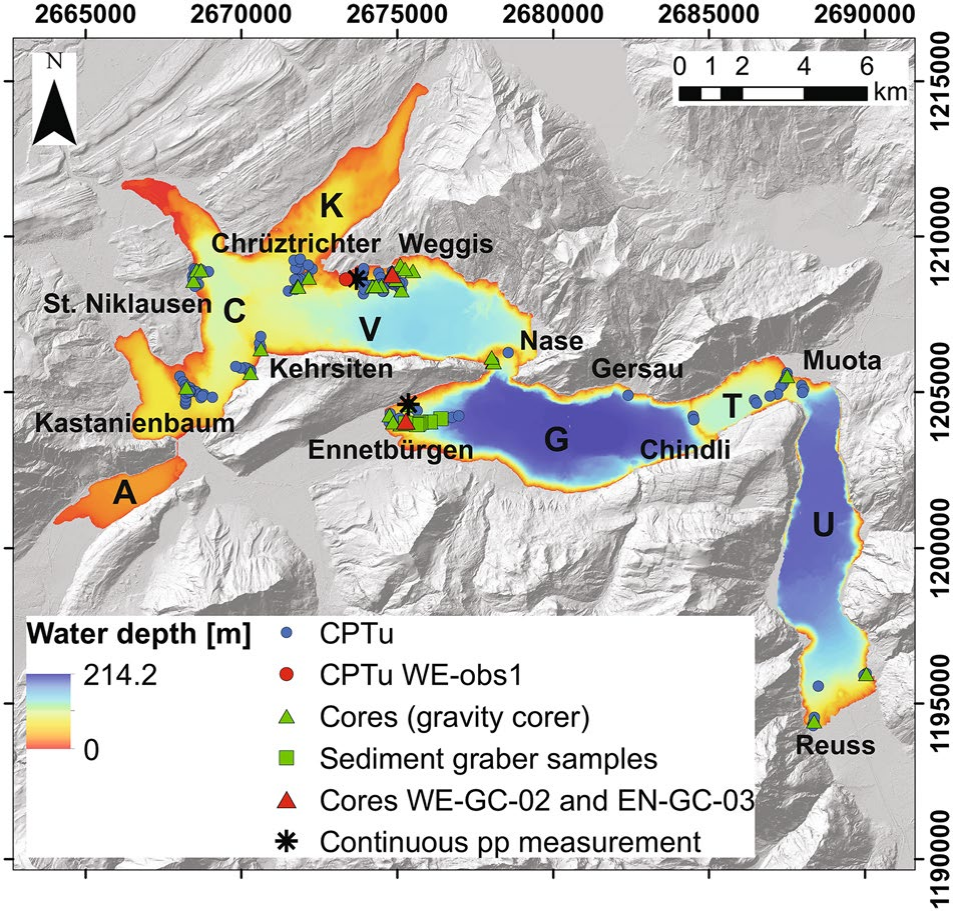
Locations of performed geotechnical investigations and names of the sites of interest: CPTu (blue and red circles) and sediment coring/sampling sites (green triangles and squares; Shynkarenko et al. 2018, 2022; Stegmann et al. 2019) on top of the bathymetric and DEM maps (Source: Federal Office of Topography swisstopo). The red circle shows the location of CPTu measurement WE-obs1 and the red triangles show the locations of the gravity cores WE-GC-02 and EN-GC-03 presented in the following chapters. Black asterisks at Weggis and Ennetbürgen show the locations of additional continuous pore water pressure (pp) measurements. The coordinates are given in the Swiss Coordinate System LV95 with units of m. Letters mark the 7 sub-basins of the Lake: A—Alpnach, C—Chrüztrichter, G—Gersau, K—Küssnacht, T—Treib, U—Uri, V—Vitznau

We selected eleven sites in six sub-basins and performed several measurements at each site (Fig. 2; the Alpnach basin is excluded from our analysis as its slopes are generally gentler than 5° and are not prone to massive failures). The selected sites are Chindli, Chrüztrichter, Ennetbürgen, Gersau, Kastanienbaum, Kehrsiten, Muota, Nase, Reuss, St. Niklausen and Weggis. In general, measurement sites are characterized by 0.1 to 15 m thick unconsolidated sediment cover. Most of the measurement locations are close to reflection seismic lines and/or sites with seismological observations with OBS (Shynkarenko et al. 2021, 2022; Lontsi et al. 2022). The slope angle is usually between 0° and 15°, rarely up to 25°. In addition to the measurements at the unfailed parts of the slopes with the undisturbed sediment cover, we performed also the measurements along the slope-perpendicular transects to get a better understanding of the sediments’ properties at different morphological parts of the slopes (e.g., undisturbed plateau, failed terrace, basin; Appendix 5 in ESM). Except for a few CPTu measurements contributing to the slope-perpendicular transects, we excluded the basin part of the lake from the analysis (areas located below the toes of the slopes and usually with slope gradient < 5°) as this zone is not prone to failures.

## Methods

**Fig. 3:**
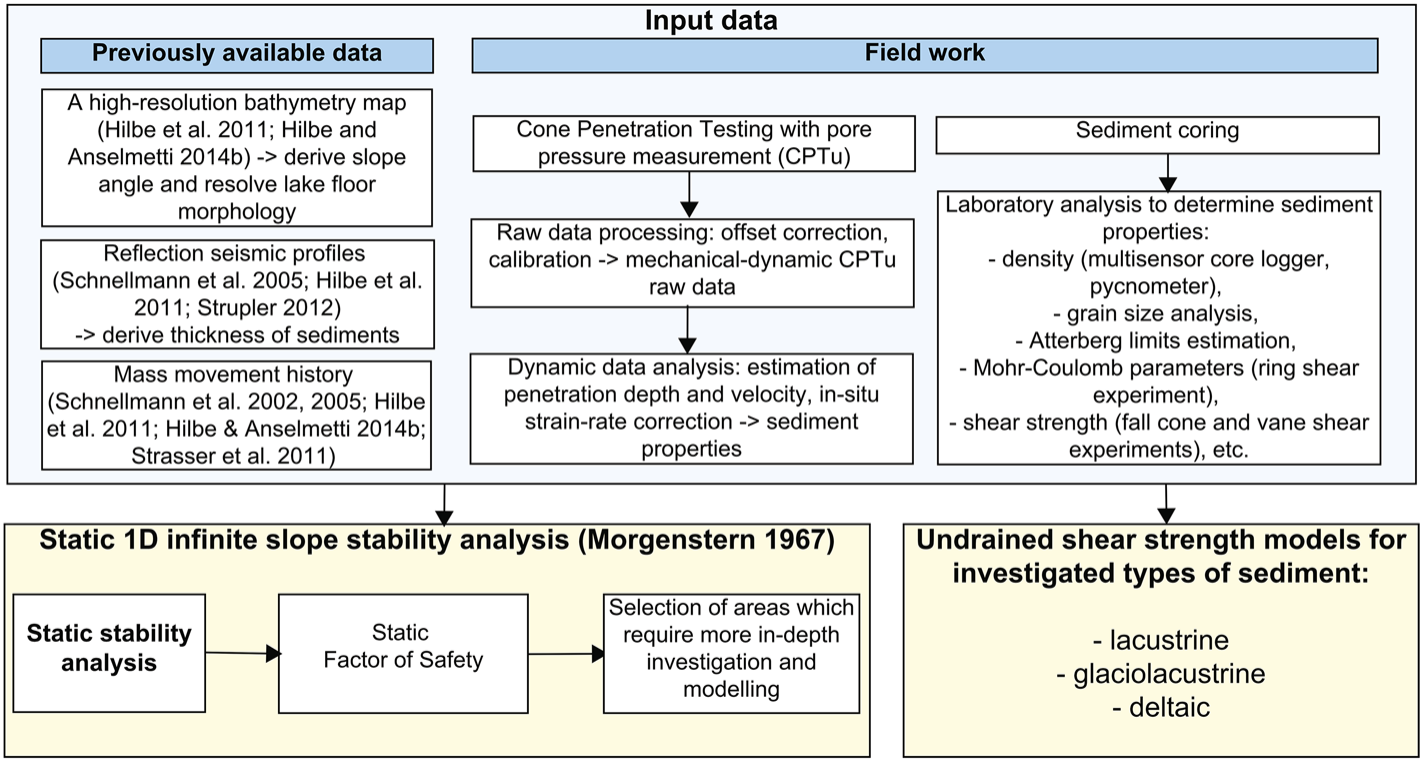
Schematic representation of the workflow used in this study to characterize the sediments and assess the stability of subaqueous slopes in Lake Lucerne

To characterize the properties, distribution and stability of lake sediments, we follow a workflow schematically presented in Fig. 3.

### Morphometric analysis of the lake floor

The morphometric analysis of the lake floor was primarily based on the processing and interpretation of the previously available datasets (Sect. 2.2). This analysis included the calculation of the slope gradient map for the lake floor, the identification of the failure scars, and the morphological classification of the slopes (e.g., to define undisturbed slopes, disturbed/failed slopes, basins, deltas taking into account the sediment properties, disturbance and occurrence of the failures in the past). This information helps to better understand the variability of sediment properties on the slopes and to identify the locations with possible sediment disturbance or missing (failed) sediment packages and, thus, is crucial for selecting sites for slope stability analysis. It is important to note that for the positioning of most of the measurements presented in this work, we used a Garmin GPS device with a positioning error of 5 m. To account for this error and the drift of the boat and measuring devices due to the currents and winds, the spatial resolution selected for the following analysis is 10 m × 10 m.

The slope angle was derived from the bathymetric map using the ArcMap 10.5.1 software (ESRI, Inc.). The thickness of sediment cover on the subaqueous slopes was retrieved via the interpretation of the homogenously processed reflection seismic data (band-pass filtered between 200–800 Hz; Hilbe et al. 2011) using Kingdom Suite 2015 software (IHS Markit, Ltd). The interpretation was only performed for the slopes potentially prone to failures, i.e., excluding the basin areas below the toe of the slope and the slopes steeper than 25°. The major part of deltaic sites was also not interpreted due to the presence of gas in the sediment and limited penetration of seismic signal. To derive the sediment thickness in deltaic areas, other techniques can be used (e.g., drilling or offshore seismological investigations; Shynkarenko et al. 2021). Three prominent horizons were traced on seismic profiles on the lateral slopes: the lake floor, the bottom of the fine-grained lacustrine sediments and the bottom of the glaciolacustrine sediments. The thickness of the lacustrine and glaciolacustrine sediments was derived via a time-to-depth conversion using an average ${v}_{p}$ velocity of 1480 m/s following previous studies (Strasser et al. 2007; Strupler 2012; Hilbe and Anselmetti 2014b) and interpolated basin-wide between the seismic lines using a natural neighbor interpolation method. This dataset allowed us also to verify the interpretation of sediment stratigraphy profiled by the CPTu, presented below.

The morphological types of the slopes were defined based on the bathymetry map, the thickness of the sediment drape (interpolated horizons on the reflection seismic lines) and available knowledge on the properties of different sediment types (defined in Strasser et al. 2007 and Strupler 2012). The main identified subaqueous slope types are: (1) undisturbed non-deltaic slopes with two subclasses: undisturbed lateral slopes and undisturbed moraine ridge slopes, (2) failed (with clear failure scars) or disturbed (with obvious signs of disturbance, e.g., uneven surface, undulations) non-deltaic slopes with three subclasses: disturbed lateral slopes, failed lateral slopes, disturbed moraine ridge slopes, and (3) deltaic slopes (following Hilbe et al. 2011 and Hilbe and Anselmetti 2014b). Moraine ridge slopes and lateral slopes are characterized by a similar sedimentary sequence in the upper, potentially failure-prone, part that consists from top to bottom of fine-grained lacustrine and glaciolacustrine sediments. They differ in the deeper structure which is, however, not of interest for this study. Deltaic slopes cover the areas where the main sediment input comes from the rivers or mountain streams.

### Sediment sampling and laboratory testing

The sediment sampling was performed using a gravity corer (Blomqvist and Abrahamsson 1985) and sediment grabber (Håkanson 1973). Gravity cores mostly retrieved homogeneous fine-grained material, while sediment grabber allowed us to collect more heterogeneous and coarse-grained sediment (e.g., from deltaic slopes), which could not be sampled with the gravity corer. The maximum sampled depth was 150 cm with the gravity corer while the sediment grabber allowed sampling of the surficial sediments only (ca. upper 5–10 cm). In total, we sampled 49 locations (42 with the gravity corer and 7 with the sediment grabber) in the lake (Fig. 2).

In the laboratory, we measured the geotechnical index properties of sediment samples from the potentially unstable slopes. In particular, we investigated the fine-grained lacustrine, glaciolacustrine and glacial sediments from the lateral and moraine ridge slopes, and fine- and coarse-grained deltaic sediments from the deltaic slopes (these lithological units were defined in Sect. 2.1). First, we performed nondestructive measurements of density and compression wave velocity for the gravity cores using a Geotek Multisensor Core Logger (MSCL; Geotek Ltd. 2021). Then, the gravity cores were split for sampling to perform the second part of the tests. Sediment samples were prepared following the standard practices (e.g., DIN 1997, 2002 or ASTM 2010). Bulk and grain densities were measured on discrete sediment samples using a Quantachrome 5200e pycnometer. Grain size analysis was performed with the laser diffractometer Beckman Coulter LS 13 320 Particle Sizing Analyzer (with resolved grain size range between 0.4 μm–2 mm; see Agrawal et al. 1991; Loizeau et al. 1994). The mineral composition of sediment samples from the lateral hemipelagic slopes was determined with an X-ray diffraction analysis (Mitchell and Soga 2005). Natural water content and Atterberg limits (DIN 1997; ASTM 2010) were measured to determine the plasticity of the sediments. Mohr–Coulomb parameters (friction angle and cohesion intercept) were derived from ring shear testing of sediment specimens (Bishop et al. 1971; DIN 2002; Stegmann and Kopf 2014). Where possible, we used the same representative samples of each lithology for different types of analysis. To support the CPTu analysis, the undrained shear strength of the sediment was measured with the fall cone and/or vane shear, while the sediment consolidation state was derived from oedometer testing. Detailed explanations for laboratory experiments (experimental setups and investigated parameters) can be found in Shynkarenko et al. (2022).

The performed laboratory testing provided us with the sediment properties required for the processing and verification of CPTu data, estimation of the sediment thickness on the slopes, and slope stability analysis (i.e., density, consolidation state, compression wave velocity, and undrained shear strength). Additionally, the measured geotechnical data allow us to compare the analyzed sediment with lithological units found in other lakes and thus, to justify an application of the ${s}_{u}(z)$ models developed below.

### Cone penetration testing with pore pressure measurement (CPTu)

The Cone Penetration Tests with pore pressure measurement (CPTu) were performed using MARUM free-fall shallow-water dynamic CPTu that includes an industrial 15 cm^2^ piezocone. The CPTu was deployed in the dynamic mode, i.e., it was dropped from the boat to the lake floor and penetrated the sediment under its weight (Stegmann 2007; Steiner 2013). In an industrial standard, CPT measurements are performed with a static penetration velocity (ca. 2 cm/s, Lunne et al. 1997; Robertson 2016). However, in the lake environment, it is quite challenging to perform static experiments, and the dynamic ones represent a logistical- and time-efficient alternative to them. In our measurements, the CPTu velocity was up to 8 m/s depending on the water depth and expected stiffness of the subsurface sediment: at the sites with expected stiff subsurface (e.g., deltas or moraine ridges), the CPTu was deployed from a smaller height and with manually-controlled velocity to avoid damaging the device.

In total, we performed 152 CPTu measurements in the lake (Fig. 2). Some of the investigated sites in Vitznau, Chrüztrichter and Küssnacht sub-basins had a few single-point CPTu measurements performed in the past (using a free-fall device similar to the one used in this study but deployed with slower velocities; Strasser et al. 2007, 2011). For the sub-basins Gersau, Treib and Uri, our measurements provide the first in situ geotechnical data. Due to the limited length of the CPTu device, the penetrated sediment depth was up to 7.4 m, depending on the thickness, grain size, and stiffness of unconsolidated slope sediments.

The parameters directly measured by the CPTu include the cone resistance ($${q}_{c})$$, sleeve friction ($${f}_{s}$$), pore water pressure behind the cone ($${u}_{2}$$), tilt, and acceleration. Due to the fast penetration of the CPTu in the sediment, the pore water filter did not have time to fully saturate/release the water, thus the absolute values of the $${u}_{2}$$ can deviate from the actual ones. The dynamic type of the conducted experiment requires a back-calculation of the penetrated sediment depth (as a product of the second integration of measured acceleration) and a correction for the nonstationary penetration rate to bring the data to the quasi-static state. Detailed information on the CPTu data processing procedure can be found in Steiner (2013), Roskoden (2015), and Shynkarenko et al. (2022). Below we summarize the processing workflow:

1. The offsets are removed from the sensor records and the raw data (in mV units of electric potential) are converted to the physical equivalents of measured parameters.
2. The penetrated sediment depth is estimated via the second integration of the acceleration record: start and stop times of penetration are identified based on the cone resistance, pore pressure and acceleration records. Estimated penetration depth is verified with the field protocols where the visually observed height of sediment leftovers on the instrument is recorded and by the comparison with reflection seismic data.
3. Penetration rate correction is applied to convert the dynamic data to a quasi-static state. Soil-specific factors and equations are selected based on the previous knowledge of the sediment and verified by the laboratory testing (Dayal and Allen 1975; Steiner 2013; Steiner et al. 2014). We use the arcsinh law and soil factors after Steiner et al. (2014), and empirical cone factor ${N}_{kt}$ with an average value of 15 (possible range is 12–20;Lunne et al. 1997; Lunne 2012; Steiner 2013; Strupler et al. 2017; Sammartini et al. 2021).
4. Undrained shear strength ${s}_{u}$ of the sediment is estimated as
  1 $$s_{u} = \frac{{\left( {q_{t} - \sigma_{v0} } \right)}}{{N_{kt} }}$$
  where ${q}_{t}={q}_{c}+{u}_{2}(1-a)$, $a=0.6$ is the net area ratio of the cone, and ${\sigma }_{v0}$ is total vertical stress (${\sigma }_{v0}=\gamma z$, where $\gamma$ is sediment unit weight and $z$ is the depth below the lake floor). Estimated ${s}_{u}$ values are verified by the comparison with the results of laboratory core testing (vane shear and fall cone experiments, see Shynkarenko et al. 2022) and previously available datasets (Strasser et al. 2007, 2011; Sammartini et al. 2021).
5. To avoid the impact of instrument self-noise and thin sediment layers on the interpretation of the ${s}_{u}$ profile, we smooth the obtained ${s}_{u}$ profile with Gaussian smoothing method for a window length of 25 cm (window length was selected after testing windows between 5 and 100 cm; in the following section we refer to this smoothed profile as to “raw” or “experimental” ${s}_{u}$ profile). This smoothing allows us to keep the main features of the data and to remove the small-scale features in the shear strength profile.

After data processing, we defined the sediment lithological units penetrated by CPTu based on absolute values of the ${s}_{u}, {q}_{t}$ and ${f}_{s}$ and their trends with depth, and verified them by the comparison with the interpreted reflection seismic profiles (see e.g., Strasser et al. 2011; Stegmann et al. 2016; Hammerschmidt 2019) and available sediment cores. Additionally, a comparison of the ${s}_{u}$ profiles and ${s}_{u}/{\sigma }_{v0}^{^{\prime}}$ ratio (where ${\sigma }_{v0}^{^{\prime}}={\sigma }_{v0}-u$ is effective vertical stress and $u$ is the pore water pressure) allowed the determination of consolidation state of the material and better differentiation of the lithological units: ${s}_{u}/{\sigma }_{v0}^{^{\prime}}>0.2$ corresponds to the underconsolidated (UC) sediments, $0.2{<s}_{u}/{\sigma }_{v0}^{^{\prime}}<0.3$ to the normally consolidated (NC) sediments, and ${s}_{u}/{\sigma }_{v0}^{^{\prime}}>0.4$ to the overconsolidated (OC) sediments (Lunne et al. 1997; Steiner 2013).

### Derivation of the empirical models for depth-dependent undrained shear strength

Using the outcome of the analysis mentioned in the previous sections, we derived the empirical models which relate the ${s}_{u}$ values estimated from CPTu data for different sediment types to the depth below the lake floor $z$. For this, we tested two functional forms of the models:

1. a power-law relation (${s}_{u}=\gamma {z}^{\alpha }$), reflecting the behavior theoretically derived for unconsolidated granular sediments (Gassmann 1951; Johnson 1985; Walton 1987), and verified by laboratory experiments (e.g., Bodet et al. 2010; Bergamo et al. 2014) as well as in situ measurements (Bachrach et al. 1998, 2000; Gofer and Bachrach 2012; Bergamo and Socco 2016);
2. a linear relation ($${s}_{u}=a+bz)$$ generally used in literature to model the depth-dependence of *s*_*u*_ in subaqueous sediments (Bartetzko and Kopf 2007; Suzuki and Yasuhara 2007; Strasser et al. 2011).

After the tests (Sect. 4.4, Appendix 2 in ESM), we selected the power-law relation and used it to derive the ${s}_{u}(z)$ models which best represent the experimental profiles for the lacustrine, glaciolacustrine and deltaic sediments. Glacial sediments were excluded from this analysis because only 24 CPTu penetrated this lithological unit, and usually, they covered just the upper 10–30 cm of the glacial layer. Deltaic sediments were considered all together to derive an ${s}_{u}(z)$ model. As a first step, we extracted and analyzed the ${s}_{u}(z)$ information from each CPTu. Each lithology was then treated separately. If one CPTu crossed more than one sediment lithotype, it contributed to the analysis of each of them. Plots for each site and summary plots for each lithology were inspected to remove outliers due to e.g., small penetration depth, disturbance of sediment, presence of erratic spikes, or overestimated strength due to the fast penetration and/or liquid-like texture of the surficial sediments. Then, we derived ${s}_{u}(z)$ curves representing the average, median, standard deviation, and 16th and 84th percentiles for each lithology. Finally, these curves were fitted with a power-law ${s}_{u}(z)$ relation.

### Static one-dimensional infinite slope stability analysis

To obtain estimates of static stability at studied locations, we applied an infinite slope analysis (Morgenstern 1967; Strasser et al. 2011; Strupler et al. 2018a). In this analysis, the factor of safety (FS) at each location is the ratio between the shear-resisting forces and the shearing forces acting on the slope:

2 $$FS = \frac{{s_{u} }}{{\sigma_{v0}^{^{\prime}} \sin \alpha \cos \alpha }}$$

The input parameters are derived from the CPTu and core analysis (undrained shear strength ${s}_{u}$ and vertical stress ${\sigma }_{v0}$) and from the bathymetry map of the lake floor (slope angle $\alpha$). To derive the effective stress (${\sigma }_{v0}^{^{\prime}}$), we assumed a hydrostatic pore water pressure $u$ inside the sediments, where $u = \gamma_{w} z$ and $\gamma_{w}$ is the unit weight of water. This inference was based on the CPTu data and two additional pore water pressure measurements (see Fig. 2 for the locations), which showed quasi-hydrostatic pore water pressure inside the tested sediments.

According to Eq. (2), as soon as the shearing stress acting along the potential sliding surface becomes equal or larger than the shearing resistance ($FS \le 1$), the slope becomes unstable (Scott and Zuckerman 1970; Baraza et al. 1990). In this study, we did not consider the possible contribution from an external seismic trigger and stick only to the static case. This decision was made due to the additional complexity of accounting for the earthquake impact in such stability analysis which might be considered in a further step of slope stability assessment. In particular, the relationship between the seismic coefficient (e.g., the fraction of PGA which acts on the sediment) used in dynamic analysis and the real earthquake-triggered ground motion is still debated (see e.g., Hynes-Griffin and Franklin 1984; Kramer 1996; Melo and Sharma 2004), especially in the case of highly-water saturated lake sediments. Such sediments cause large amplification of seismic waves and are expected to respond nonlinearly during strong seismic shaking.

## Results

### Morphometric analysis of the lake floor

**Fig. 4:**
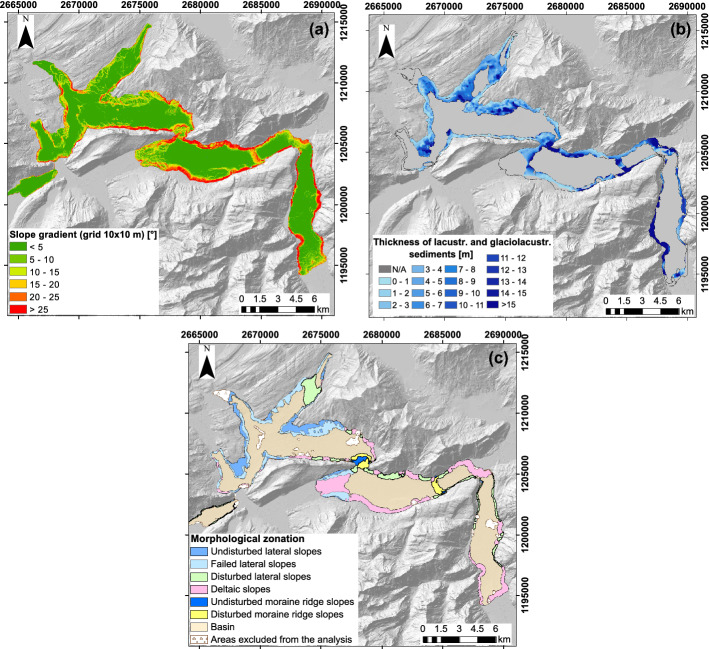
**a** Slope gradient map for Lake Lucerne on the scale of 10 m × 10 m. **b** Cumulative thickness map for the lacustrine and glaciolacustrine sediments. **c** Morphological zonation of the lake floor depending on the subsurface structure, slope gradient and sediment disturbance. The coordinates are given in the Swiss Coordinate System LV95 with units of m

We extracted the slope gradient map of the lake floor (Fig. 4a) and the thickness of the lacustrine and glaciolacustrine sediments (Fig. 4b) interpolated on a 10 m × 10 m grid. Due to the variable distance between the reflection seismic profiles, the areas with less data coverage might have interpolation artefacts in the thickness maps. We identified also different morphological zones on the lake floor as described in Sect. 3.1 (Fig. 4c). The results of the morphometric analysis represent the base for the investigation site selection. Additionally, the CPTu measurements and sediment coring were carried out at the locations with good seismic data coverage, except for deltaic slopes, and thus can rely on the interpreted seismic profiles and interpolated sediment thickness maps.

### Sediment sampling and laboratory testing

**Fig. 5:**
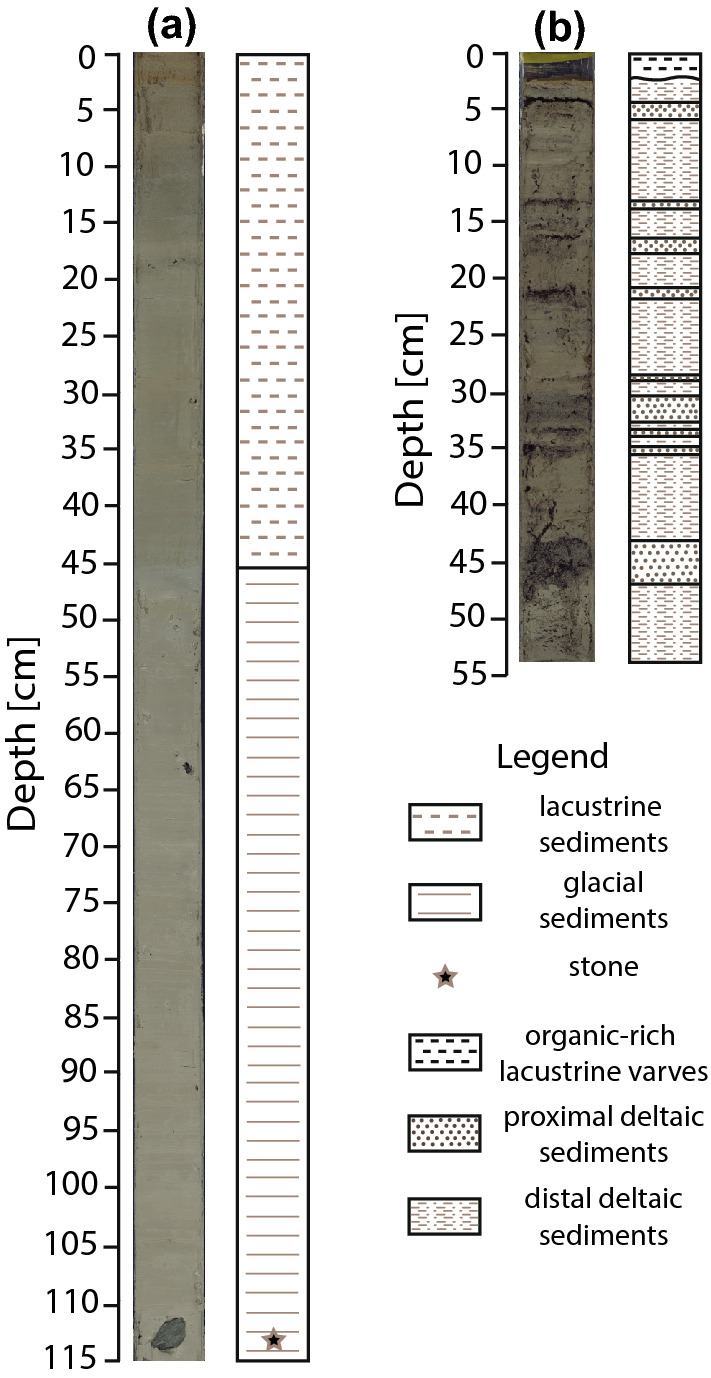
Examples of sediment cores (photos and geological interpretation) retrieved at **a** lateral slope, core WE-GC-02; a stone can be observed inside the glacial sediments (between 110 and 115 cm depth) and **b** deltaic slope, core EN-GC-03. See Fig. 2 for the core locations

The retrieved sediment cores provided us with the samples of lacustrine, glaciolacustrine, glacial and fine- and coarse-grained deltaic sediments (as defined in Sect. 2.1), which were then tested in the laboratory. The cores retrieved at the lateral and moraine ridge slopes mostly contain homogeneous (in terms of the grain size) sediments (Fig. 5a), while the cores from the deltaic slopes have very heterogeneous sediment sequences with variable grain size distributions (Fig. 5b). Information obtained from the lithological description and laboratory testing of the sediments allowed us to better differentiate between the investigated sediment lithologies, obtain input to the stability analysis (sediment unit weight), and verify the interpretation of the CPTu and reflection seismic data.

Table 1 summarizes the main parameters determined for each lithology from laboratory testing. Detailed results of the laboratory tests and core photos can be found in the report compiled by Shynkarenko et al. (2022). The lacustrine sediments are described as homogeneous silty clays of intermediate to extremely high plasticity (ASTM 2017; BS 5930,2020) according to their Plasticity Index (PI = 71–75%), Liquid Limit (LL = 95–115%), Plastic Limit (PL = 24–40%) and grain size distribution. These sediments are typically slightly underconsolidated to normally consolidated and have unit weight in the range of 12–15 kN/m^3^_._ The glaciolacustrine sediments with PI = 32%, LL = 50%, and PL = 18% are described as thinly-laminated clays and clayey silts of low to intermediate or high plasticity. Similar to the lacustrine sediments, glaciolacustrine ones are slightly underconsolidated to normally consolidated. Their unit weight typically lays in the range 14.5–16.5 kN/m^3^. The glacial sediments have PI = 23–30%, LL = 37–45%, and PL = 14–16% and are represented by thinly-laminated clayey silts to silty clays of low to intermediate plasticity which are often deformed. Gravels and sparse sand material are quite often found in the glacial sediments. Deltaic sediments mostly stay between the sandy silts and silty sands and have variable grain size distribution and unit weight. Following the same strategy as in Shynkarenko et al. (2021), we define two end-members (sublithologies) of deltaic sediments found at Muota, Ennetbürgen and Reuss sites (Fig. 2). The first one is probably related to the distal deltaic sedimentation and consists of fine-grained clayey silts and silty clays with low sand content. The second group is attributed to the proximal deltaic or flood sedimentation as it is more coarse-grained and consists mainly of a heterogeneous sequence of silty sands.

**Table 1:** Geotechnical parameters of the analyzed sediments

Sediment lithology	Grain size distribution			Consolidation state, ${s}_{u}/{\sigma }_{v0}^{^{\prime}}$ [–]	Unit weight [kN/m^3^]	Atterberg limits			Mohr–Coulomb parameters	
	Clay [%]	Silt [%]	Sand [%]			Plasticity index [%]	Liquid limit [%]	Plastic limit [%]	Cohe-sion [kPa]	Friction angle [°]
Lacustrine	40–60	35–50	0–10	Slightly UC-NC	14 (possible range 12–15)	71–75	95–115	24–40	0.6	30
Glaciolacustrine	40–60	35–50	0–10	Slightly UC-NC	15.5 (14.5–16.5)	32	50	18	2.1	22
Glacial	40–60	35–50	0–10	OC	18 (16.5–20)	23–30	37–45	14–16	1.9	22
Distal deltaic (silt content > 40%)	15–30	40–65	10–30	–	~ 16.5 (12–21)	–	–	–	0	29
Proximal deltaic (sand content > 50%)	5–15	20–40	50–70	–	~ 16.5 (12–21)	–	–	–	0	30

*UC* underconsolidated, *NC* normally consolidated, *OC* overconsolidated, “–” means that no laboratory testing was performed to measure the corresponding parameter

All tested sediments have relatively high water content: 150–190% for non-deltaic sediments, and 25–80% for deltaic sediments. With depth, the water content decreases. X-ray diffraction analysis did not reveal any major difference in the composition of the lacustrine, glaciolacustrine and glacial sediments which could lead to the differences in their mechanical behavior. Thus, possible differences in the stability of these sediments can be related to different sedimentation rates and conditions (e.g., water depth, distance from the shore, slope angle). An average compression wave velocity of the sediment required for the time-depth conversion of reflection seismic profiles was measured with the MSCL and is around 1480 m/s.

### Cone penetration testing with pore pressure measurement (CPTu)

**Fig. 6:**
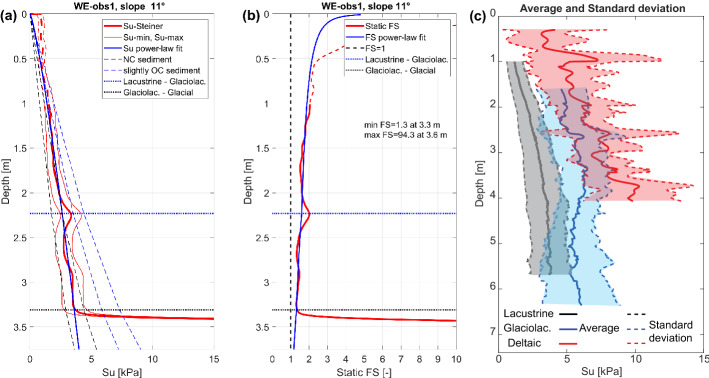
**a** Undrained shear strength $s_{u}$ profile for the CPTu WE-obs1 (see Fig. 2 for location). The red lines show the average $s_{u}$ profile (thick line, $N_{kt} = 15$) and its possible variation for the upper and lower limits of $N_{kt}$ (thin lines, $N_{kt} = 20$ and $12$, respectively). The continuous blue line shows the power-law fit of the $s_{u}$ profile. The upper 1 m of the profile is characterized by overestimated shear strength and should not be used for any interpretation. The black and blue dashed lines show the $s_{u}$ ranges for the normally consolidated (NC) and overconsolidated (OC) sediments, respectively. The dotted blue and black lines show the boundaries between the sediment units. **b** Factor of Safety FS for WE-obs1 measurement point: the red line shows an estimate based on the average experimental $s_{u}$ profile, the blue line shows the FS estimated for the power-law fit of the $s_{u}$ profile. The vertical dashed black line shows the FS = 1, which separates the statically stable (FS > 1) and unstable (FS ≤ 1) states of the slope. **c** overlap of the average $s_{u}$ profile and corresponding standard deviation for the lacustrine, glaciolacustrine and deltaic sediments

For each performed CPTu measurement, the undrained shear strength ${s}_{u}$ was derived as a function of penetration depth $z$(i.e., depth below the lake floor) showing us the development of the sediment strength with depth at each location and its possible lateral variations. An example of the ${s}_{u}$ profile from the undisturbed lateral hemipelagic slope at Weggis (CPTu WE-obs1, Fig. 2) is presented in Fig. 6a. The red thick line “${s}_{u}$-Steiner” represents the experimental ${s}_{u}$ profile estimated using the arcsinh law and soil factors after Steiner et al. (2014), and empirical cone factor ${N}_{kt}=15$. Thin red lines (${s}_{u}$-min and ${s}_{u}$-max) show the effect of ${N}_{kt}$ values which can be used for the ${s}_{u}$ calculations; minimum ${s}_{u}$ values correspond to ${N}_{kt}=20$ and maximum – to ${N}_{kt}=12$. The blue line exemplifies the fit of the experimental ${s}_{u}$ profile with a power-law relation. The ${s}_{u}$ ranges that correspond to the normally consolidated and slightly overconsolidated sediment are shown with the black (“NC sediment”) and blue “slightly OC sediment” dashed lines, respectively. The horizontal blue and black dotted lines (“Lacustrine-Glaciolac.” and “Glaciolac.-Glacial”, respectively) show the boundary between the corresponding lithological units. Figure 6b shows the calculated Factor of Safety (FS, see Sect. 4.5), both for the measured ${s}_{u}$ profile (thick red curve) and ${s}_{u}$ profile fitted with a power-law relation (thick blue curve). The vertical black dashed line shows the boundary between the stable and unstable state of the location (FS = 1). FS values are all above 1 for this site. Examples of the ${s}_{u}$ profile and FS variation with depth at deltaic and moraine ridge slopes can be found in Appendix 1 in ESM (Figs. 10 and 11).

Each ${s}_{u}(z)$ profile was interpreted to identify the profiled sediment-lithological units. At the presented site WE-obs1, the measurement crossed three units: lacustrine, glaciolacustrine and glacial (Fig. 6a and b). All CPTu performed at non-deltaic slopes profiled the lacustrine sediments. The bottom of the glaciolacustrine and glacial sediments normally found below the lacustrine layer was not always reached due to the limited length of the measurement device and increasing sediment stiffness with depth.

Based on the derived ${s}_{u}$ profiles (e.g., Fig. 6a), the lacustrine sediments can be characterized as slightly underconsolidated to normally consolidated, their ${s}_{u}$ is gradually increasing with depth. The glaciolacustrine sediments are also characterized as slightly underconsolidated to normally consolidated, although their ${s}_{u}$ is increasing with depth with a lower gradient than observed for the lacustrine sediments or even stays nearly constant. This change in the gradient can be seen in Fig. 6a. Spikes in the ${s}_{u}$ profiles are quite rare for these two sediment lithotypes and are probably related to the isolated pebbles or shells. Glacial sediments appear to be overconsolidated and are characterized by the presence of spikes in the ${s}_{u}$ profiles, well seen in Fig. 6a. Deltaic sediments show quite heterogeneous ${s}_{u}$ profiles with many spikes (Appendix 1, Fig. 11a in ESM; Appendix 3, Fig. 13c in ESM). An overlap of the average ${s}_{u}$ profile and corresponding standard deviation for the lacustrine, glaciolacustrine and deltaic sediments is shown in Fig. 6c. A clear distinction in terms of ${s}_{u}$ trends and values distribution can be seen between these lithologies that proves the need to describe each of them with a separate functional model. Glacial sediments are not shown due to the limited number and penetration depth of the measurements inside this layer.

The appearance of ${s}_{u}(z)$ profiles can also vary between the previously defined types/parts of slopes (Fig. 6a; Appendix 1, Figs. 10a and 11a in ESM; Appendix 3, Figs. 14, 15, and 16 in ESM). For example, the ${s}_{u}$ values and trends for the sediments deposited on the lateral hemipelagic and moraine ridge slopes are quite consistent, implying a homogeneous sediment cover at these sites. However, the thickness of the sediment on these slopes may vary depending on the water depth, slope angle etc. Deltaic slopes bear quite a heterogeneous sediment cover which, as mentioned in Sect. 4.2, can be divided into two end members: fine-grained material related to the distal deltaic sedimentation and coarse-grained sediment related to the proximal deltaic sedimentation or flood deposits.

An important observation in most of the CPTu profiles is the overestimation of the undrained shear strength in the upper part (down to 0.3—1 m). This overestimation is caused by the high impact and penetration velocity of the CPTu (Morton 2015) and the viscous-liquid-like texture of the surficial sediments which can lead to uncertainties in the estimated penetration depth. Overall, 10 CPTu measurements with the length almost equal to or less than the thickness of the overestimated part of the ${s}_{u}\left(z\right)$ profile were excluded from the further analysis. In the rest of the CPTu profiles, we mark the unreliable (overestimated) part of the ${s}_{u}$ profile with a dashed line (Fig. 6a; Appendix 1, Figs. 10 and 11 in ESM).

### Derivation of the empirical models for depth-dependent undrained shear strength

We tested two functional forms (power-law and linear relation) to fit the ${s}_{u}(z)$ profiles. Both of them provided a similar fit to the data (Appendix 2, Fig. 12 in ESM). However, the power-law relation allows us to avoid an overestimation of the undrained shear strength at a large depth. In the following, we only use the power-law relation to fit the ${s}_{u}\left(z\right)$ data. Some examples of the fit and corresponding FS curves are given in Fig. 6 and Appendix 1, Figs. 10 and 11 in ESM (blue solid curves).

**Fig. 7:**
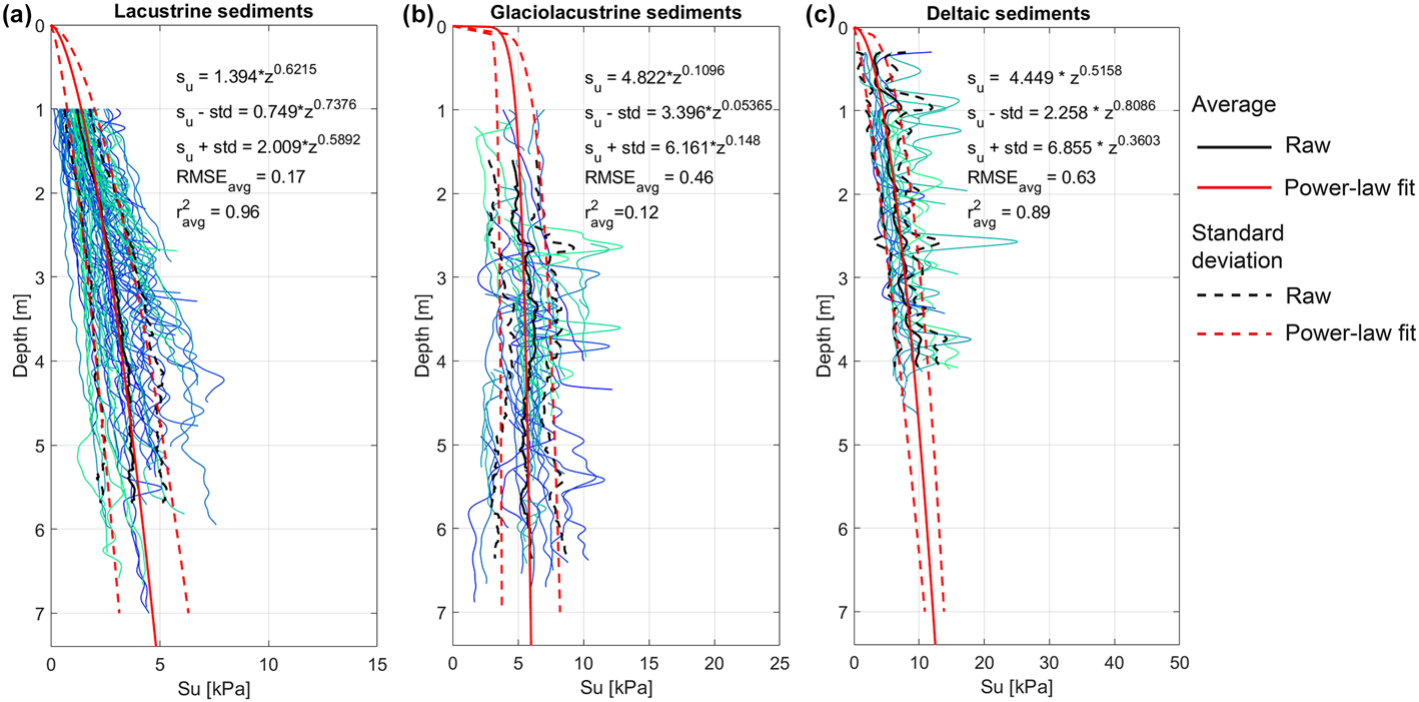
Undrained shear strength profile (average and standard deviation) for **a** lacustrine, **b** glaciolacustrine, and **c** deltaic sediments fitted with the power-law equation (the equations, RMSE and r^2^ are shown on the plots)

To derive the generalized ${s}_{u}(z)$ models for the lacustrine, glaciolacustrine and deltaic sediments, we first inspected all CPTu that crossed these sediments and removed the outliers. The outliers include the CPTu measurements with too short penetration depth (< 0.6 m), the presence of erratic spikes in the ${s}_{u}(z)$ profiles (possibly due to the large-sized pebbles and stones), or pre-existing disturbance of penetrated sediments (for the CPTu performed in the slope-basin transition zone). Then, we selected for further analysis 74 CPTu drops that profiled lacustrine sediments, 52 drops that profiled glaciolacustrine sediments, and 22 drops that profiled deltaic sediments (Appendix 3, Table 3 in ESM). Selected CPTu profiles for each lithology were processed together to derive the average and median ${s}_{u}(z)$ profiles with associated standard deviation and selected percentiles. Next, the derived representative ${s}_{u}$ profiles were fitted with a power-law relation. Corresponding equations are given in Table 2. Figure 7 shows all CPTu selected for the analysis of each type of sediment, average ${s}_{u}$ profile and its standard deviation and the power-law fit of the corresponding ${s}_{u}$ curves; the same plots for the median and 16^th^ and 84^th^ percentiles can be found in Appendix 3, Fig. 13 in ESM.

**Table 2:** Power-law equations representing the ${s}_{u}(z)$ relationship the fine-grained lacustrine, glaciolacustrine and deltaic sediments: average value and standard deviation bounds, median value and 16th and 84th percentiles; $z$ value is in meters below the lake floor

Lithology	Average ${s}_{u}\left(z\right)$ [kPa]	Average ${s}_{u}\left(z\right)$– std [kPa]	Average ${s}_{u}\left(z\right)$+ std [kPa]	Median ${s}_{u}\left(z\right)$ [kPa]	16th percentile of ${s}_{u}\left(z\right)$ [kPa]	84th percentile of ${s}_{u}\left(z\right)$ [kPa]
Lacustrine	$1.394{z}^{0.6215}$	$0.749{z}^{0.7376}$	$2.009{z}^{0.5892}$	$1.364{z}^{0.6055}$	$0.7667{z}^{0.6922}$	$2.11{z}^{0.5459}$
Glaciolacustrine	$4.822{z}^{0.1096}$	$3.396{z}^{0.05365}$	$6.161{z}^{0.148}$	$4.839{z}^{0.1241}$	$3.58{z}^{-0.0186}$	$6.05{z}^{0.1438}$
Deltaic (background trend)	$4.449{z}^{0.5158}$	$2.258{z}^{0.8086}$	$6.855{z}^{0.3603}$	$3.816{z}^{0.5861}$	$2.691{z}^{0.6734}$	$6.353{z}^{0.4122}$

The derived power-law models reliably resemble the shape and the trend of the experimental ${s}_{u}\left(z\right)$ curves. We also attempted to subdivide the CPTu profiles belonging to the same sediment type into sub-groups depending on the slope angle, terrain class (following the definition of Burjánek et al. 2014), and a morphological class of the slope (Appendix 3, Figs. 14, 15, and 16 in ESM). Potentially, such a more detailed classification could allow decreasing the uncertainty in the ${s}_{u}(z)$ models. Unfortunately, our attempts did not reveal any additional grouping criteria (Appendix 3, Table 4 in ESM) as all subgroups were characterized by similar ranges of ${s}_{u}(z)$ distribution with depth and similar fitting equations. We assume that our models could be improved when considering a multivariate regression where more arguments contribute to the ${s}_{u}(z)$ relationship (e.g., slope roughness, distance to the shore, water depth), but we did not explore this option in the framework of this paper.

### Static one-dimensional infinite slope stability analysis

For each CPTu point, we performed a static infinite slope stability analysis to investigate the failure potential of different locations in the lake and select the most crucial and failure-prone areas. An example of the Factor of Safety (FS) estimated for the WE-obs1 can be found in Fig. 6b. Additionally, we compared the FS estimated for the experimental ${s}_{u}\left(z\right)$ profile with the FS estimated for the power-law-fitted ${s}_{u}\left(z\right)$ profile. FS estimates for the experimental and fitted data match well. The most important part of these calculations is the minimal FS inside the sediment drape on the slope.

**Fig. 8:**
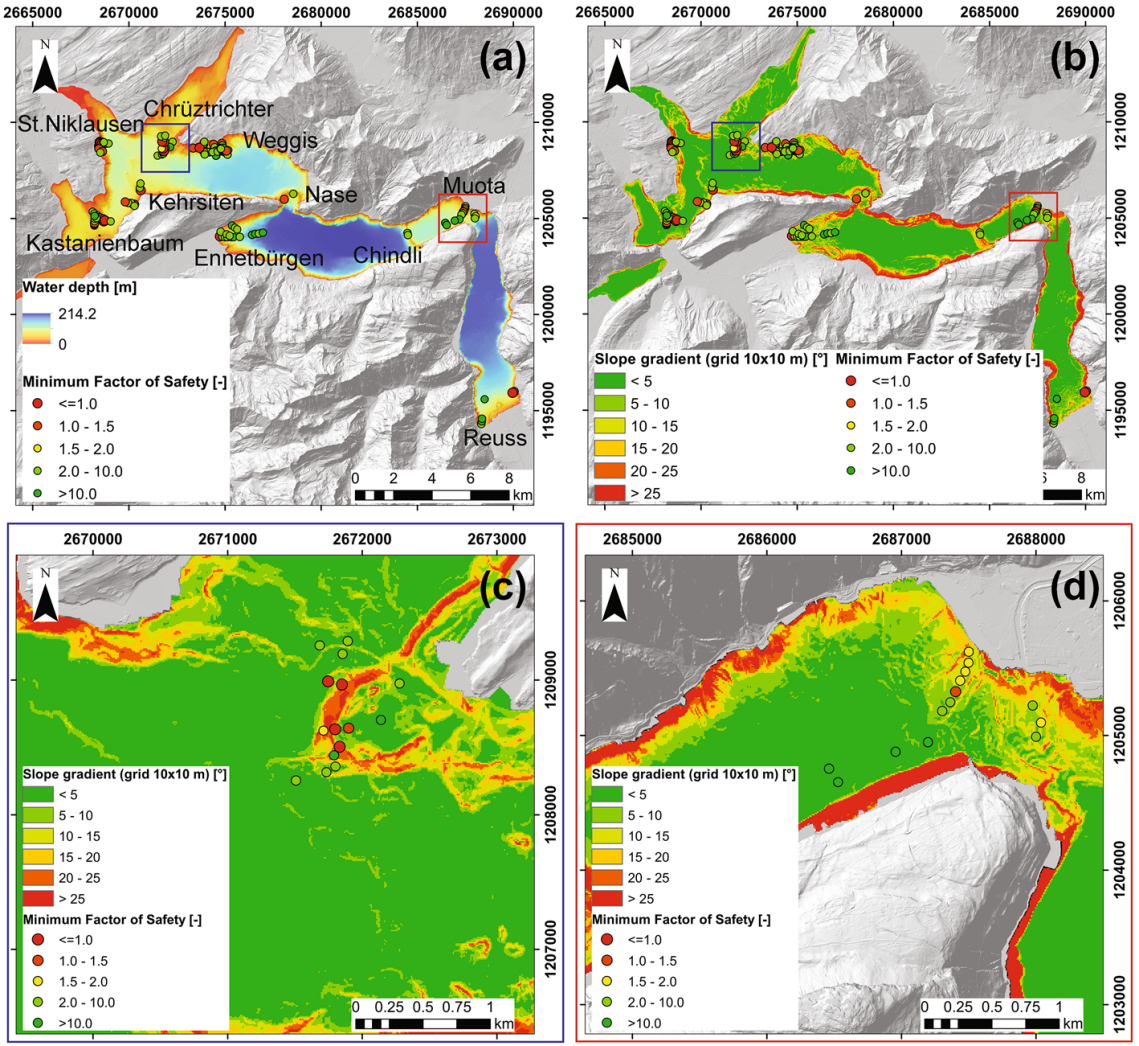
**a–b** Static Factor of Safety FS for the locations with CPTu measurement on top of the bathymetry and slope gradient maps, respectively: red dots show the statically unstable areas. **c–d** Static FS on top of the slope gradient maps for Chrüztrichter and Muota sites, respectively

Figure 8a and b shows the minimum FS estimated for each CPTu location on top of the bathymetry and slope gradient maps, respectively. Quantitative results for each CPTu can be found in Appendix 4 in ESM (Table 5). The failure plane on the non-deltaic slopes is usually inside the glaciolacustrine layer (closer to the bottom part) as it was also discussed in previous studies (Schnellmann et al. 2006; Strasser et al. 2011; Hilbe and Anselmetti 2015; Strupler et al. 2018b). For deltaic slopes, the smallest FS values correspond to the softest and most fine-grained layers in the sediment profile. For most of the sites, we observe the tendency of FS to decrease with increasing slope angle, as expressed in Eq. 2, describing the significant influence of slope angle on the relation between shearing and resistant forces acting on the slope (Fig. 8b–d, and Appendix 5 in ESM for the sites not shown on detailed maps in Fig. 8). The majority of the sites that failed in the past and that have a higher probability to fail in future have slope angles steeper than 5–10°. Additionally, deltaic sites (e.g., Muota in Fig. 8d) usually have FS > 1 and higher FS values than the non-deltaic slopes for the same slope angle due to the higher shear strength of the deltaic sediment.

Based on the stability analysis results presented in Fig. 8 and Appendices 4 and5, and taking into account the collected geotechnical data and the assessments in previous studies, we can classify the investigated sites into three categories:

1. Currently unstable areas, i.e., areas that have FS < 1. Such isolated locations are mostly found at Chrüztrichter, Kastanienbaum and St. Niklausen sites (Fig. 8). The sediments at these sites are mostly composed of clay and silt. At Kastanienbaum, we also noticed the presence of gas in the sediments, based on characteristic signatures in the seismic profiles and direct identification in the sediment cores.
2. Currently stable areas close to the unstable state (1 ≤ FS < 1.5–2) which require a more detailed and comprehensive investigation. This FS range was selected as a compromise between different approaches to separate between the stable and unstable state of the slope (Kramer 1996; Silva et al. 2008; Stark and Ruffing 2017; Herza et al. 2018; Schnaid et al. 2020). Parts of the slopes at St. Niklausen, Weggis, Kastanienbaum, and Muota sites belong to this group (Fig. 8).
3. Currently stable areas (FS > 1.5–2) for which we do not expect failure under static conditions, i.e., if no additional loading is applied to the sediment. These areas mainly correspond to the deltaic sites, i.e., Ennetbürgen, Muota, and Reuss, and previously failed or basin parts of the non-deltaic slopes.

The conditions which could potentially trigger the failure of different slope categories are discussed in the following section.

## Discussion

The collected dataset and performed data analysis provided us with the geotechnical parameters of sediments and slope stability information for different locations in Lake Lucerne. Additionally, a dense network of CPTu measurements allowed us to derive the generalized model of the undrained shear strength of failure-prone lithological units which can be transferred to other Swiss lakes. However, there are two important aspects of our study that need to be addressed: (1) despite the very detailed level of investigations, high density of measurements, and homogeneous processing and interpretation of data, the slope stability analysis is affected by different types of uncertainties as well as assumptions and simplifications introduced during the data acquisition and processing. Thus, it is important to understand the main uncertainty sources present in our data and results (e.g., Stark and Ruffing 2017). Additionally, the spatial variability of sediment properties has to be discussed; (2) to verify the applicability of our ${s}_{u}(z)$ models to other Swiss lakes with similar sedimentation history and sediment composition, they have to be compared with the ${s}_{u}(z)$ estimates from the other available investigations. Below, we address these aspects.

### Uncertainties in data and results

The first source of uncertainty in our data is unavoidably introduced during the data acquisition and is related to the error in the measurement position due to (1) the precision of used GPS device of ± 5 m, and (2) the research vessel/CPTu/coring devices drift in the water during the measurements caused by wind and currents.

The morphometric analysis of the lake floor strongly depends on the quality of the input data. The bathymetry map has a resolution of 1 m × 1 m and allowed us to derive reliable slope gradient maps on the downsampled bathymetry grid (10 m × 10 m). The quality of the thickness maps of different sediment units depends not only on the quality of seismic data but also on its spatial distribution. In the areas with poor seismic lines coverage the derived thickness maps are less reliable and might be contaminated by the interpolation artefacts. Additionally, the morphological classification of the slopes proposed within this study is non-unique and can be modified (e.g., simplified) according to the project goals. Overall, the morphometric analysis compares well with previous investigations (Strasser et al. 2011; Hilbe and Anselmetti 2015; Strupler et al. 2020).

The CPTu processing involves the application of soil-specific factors and penetration rate corrections which depend on the information available for the target sediments and experimental setup. When selecting these parameters, we relied on the experience of previous studies in Lake Lucerne and other studies for similar sediments (e.g., Lunne et al. 1997; Steiner et al. 2014; Sammartini et al. 2021), as well as on the laboratory analysis of the sediment cores. Additionally, during the field CPTu measurements, we had limited control over the penetration depth of the device and had to back-calculate it from the acceleration recording. The start and end time of the penetration is assessed by comparing the acceleration trace, and pore pressure and cone resistance logs. The resulting variability of estimated (experimental) parameters, in particular the undrained shear strength, can be up to 20–35% from the obtained value.

The derived empirical ${s}_{u}\left(z\right)$ relations can be used in any lake with similar sediments, provided the availability of basic geotechnical index properties of the sediments (e.g., unit weight, grain size, Atterberg limits) to verify the similarity between the target sediments and the ones analyzed in this study. To cover the observed variability in the properties of each sediment-lithological unit, our models provide relatively wide ranges of possible ${s}_{u}$ values. As shown with the attempt to narrow these ranges by dividing ${s}_{u}\left(z\right)$ profiles into more than three groups, improved precision of the models requires direct measurement at the site of interest and can potentially be reached by replacing univariate regression with the multivariate one which takes into account more site-specific parameters like water depth, slope morphology, subsurface structure, and sedimentation rates. The introduced ${s}_{u}\left(z\right)$ equations are valid only for undisturbed sediments and cannot be used for disturbed sediments, e.g., mass-movement deposits. We can speculate that disturbed sediments will probably have higher ${s}_{u}$ values than the undisturbed (e.g., due to the compaction) and their ${s}_{u}$ might tend to be at the upper limit of the proposed ranges.

Additionally, it is not possible to define the optimal number of measurements and measurement locations to derive the ${s}_{u}\left(z\right)$ models for other lakes in the way we did in this paper because these numbers will strongly depend on the variability of sediment properties: the less variability is observed in the parameter, the fewer measurements would be needed to describe it. For example, Figs. 14, 15, and 16 in Appendix 3 in ESM show the average ${s}_{u}\left(z\right)$ profile and its standard deviation for different groups of CPTu measurements depending on the slope morphology: we observe very similar trends for the lacustrine sediments and a bit more variability for the glaciolacustrine and deltaic sediments. Thus, a different minimum number of tests might be needed for each lithology to obtain a representative trend of ${s}_{u}\left(z\right)$ or other parameters.

The core analysis is often affected by the possible sediment disturbance caused by the changes of the environmental conditions (e.g., temperature, pressure), transportation (e.g., compaction caused by shaking) and storage (e.g., temperature, moisture). Such effects can be minimized but not completely avoided.

### Slope stability analysis

The static 1D infinite slope method described by Eq. (2) and used in this work is conservative and simplistic (as required in engineering practice). It is important to note, that it is based on the assumptions that (1) the thickness of the failure-prone sediment package is much smaller than the length of the slope; (2) the failure plane of the slope is parallel to its surface; (3) the failure-prone sediment mass acts as a rigid block. While the first two assumptions are in general well satisfied for the investigated slopes, the third one is violated.

On the one hand, such simplified analysis allows an assessment of large areas at reasonable costs. On the other hand, the method does not account for the interslice forces due to the sediment cohesion and friction, complex slope geometry, and is based on the limited information about the investigated site. Most probably, a 2D analysis accounting for the mentioned factors would also provide higher and more realistic FS values. Additionally, any uncertainty contributing to the input parameters alters the calculation result and needs to be assessed carefully. In particular, slope angle has a major impact on the FS value. The uncertainty in the CPTu or core location will lead to uncertainty in the related slope angle. We attempted to smooth this effect by downsampling the bathymetry map to 10 m × 10 m resolution. Possible variation of the undrained shear strength value due to the selection of processing parameters might lead to variations of FS. Generally, the effect is just a scaling of all FS values, so that the relative difference in the failure potential of different sites will remain. Last but not least, there is an effect of selected sediment unit weight and pore water pressure values on the FS. Although multiple measurements of sediment density confirmed the selected average values, the core density logs show a unit weight variability of up to 10–15% for the lacustrine sediments (mostly in the surficial part of the profile), up to 10% for the glaciolacustrine and glacial sediments, and up to 20–40% for the deltaic sediments. Introducing these variabilities into the calculations would affect both undrained shear strength and effective vertical stress values, and thus the FS. However, a precise prediction of the unit weight of sediments at a given location and depth is impossible without retrieving the corresponding core samples. Our assumption of hydrostatic pore water pressure was deduced from a limited number of in situ measurements. Therefore, we cannot exclude local pore pressure variations in the sediment due to e.g., the local morphology, aquifer discharge or presence of gas. However, in the absence of external perturbations, the hydrostatic pore water pressure assumption should be valid for the investigated sites as they have relatively slow sedimentation rates (non-deltaic slopes) or consist of the quite coarse-grained sediments which allow the dissipation of the excess pore pressure. It is also not possible to provide an absolute value for the possibly accumulated error in the final calculation. Additionally, the minimum factor of safety (FS) which is considered a boundary between the stable and unstable states depends on the goals of a study. Generally, it is selected between 1 and 1.5 or even more (Kramer 1996; Silva et al. 2008; Duncan et al. 2014; Stark and Ruffing 2017; Herza et al. 2018; Schnaid et al. 2020).

Taking into account the mentioned issues, we suggest considering the outcome of the presented analysis more as a qualitative rather than a quantitative assessment of slope failure potential. The identified areas of increased failure potential should be selected as targets for more comprehensive investigations in future. As we showed above, the investigated slopes can already be divided into three categories based on the estimated Factor of Safety values. For these categories, we can also discuss the potentially failure-triggering conditions. For the locations belonging to the category “(1) currently unstable areas”, we do not know any reports of the ongoing failures, so interpret the behavior of these slopes as a slow creeping of the highly cohesive and plastic material. Potentially, the repeated bathymetric mapping would allow us to prove or reject this hypothesis. Similar locations of such statically quasi-unstable areas were described by Strasser et al. (2011). They characterized these areas as the “hot spots” for the subaqueous slides as they, at least Chrüztrichter and St. Niklausen, have experienced failures in the past. In any case, past slope failures in these areas already removed a large part of the sediment cover of the slopes thus decreasing their tsunamigenic potential in case of repeated failure. The category “(2) currently stable areas close to the unstable state” includes the sites which are presently stable but are very probable to fail in case of a strong seismic event (like the 1601 Unterwalden *M*_w_ 5.9 earthquake) or due to the sediment overloading/excess pore pressure generation (like during the spontaneous Muota delta collapse in 1687). The category “(3) currently stable areas” mostly includes the deltaic sites composed of the material with high strength and in some cases, very gently inclined non-deltaic slopes (< 5°). In case of a strong seismic event, their failure cannot be excluded but is less probable than for the classes (1) and (2). However, in addition to the other issues, in the performed analysis, we did not account for possible seismic events which might substantially affect the stability of tested slopes. To get a comprehensive overview of the slope stability under different conditions, an additional analysis (e.g., using the pseudostatic LEM or the Newmark method; Newmark 1965; Kramer 1996; Jibson 2011; Duncan et al. 2014) together with the dynamic sediment testing and physical modelling are needed.

### Comparison with geotechnical data previously derived in Swiss lakes

**Fig. 9:**
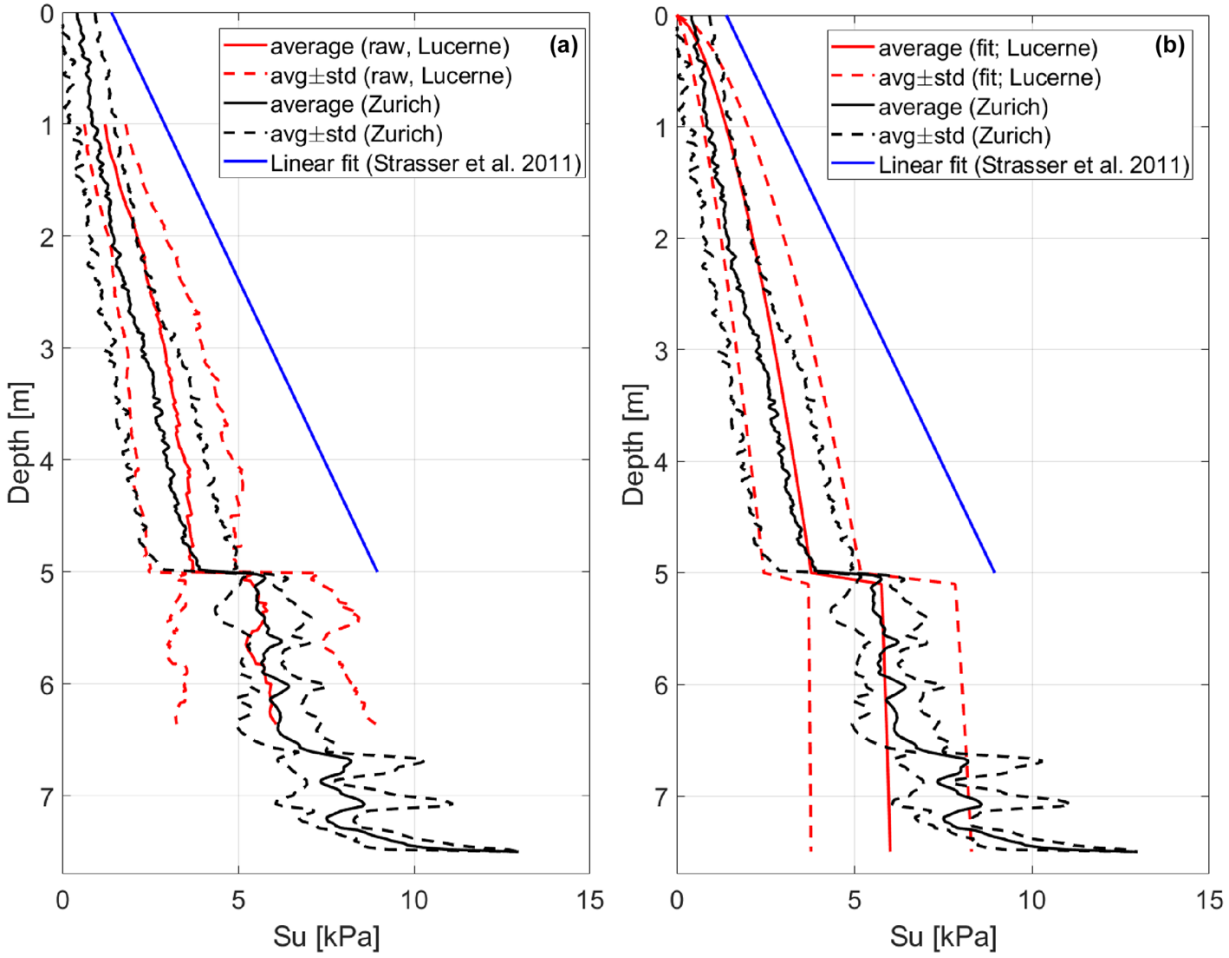
Comparison of the average undrained shear strength profile derived for Lake Zurich (black lines, Strupler et al. 2017), and linear trend of $s_{u} \left( z \right)$ proposed for lacustrine sediments in Lake Lucerne by Strasser et al. (2007; blue line) with: **a** average measured undrained shear strength profile and **b** power-law fit of the average $s_{u} \left( z \right)$ profile for Lake Lucerne derived in this study. The upper 5 m of the profile consist of lacustrine sediments, below are the glaciolacustrine ones

To verify the applicability of our ${s}_{u}(z)$ models to other Swiss lakes with similar sedimentation history and sediment composition, we compared our empirical ${s}_{u}(z)$ profiles for the lacustrine and glaciolacustrine sediments with the average ${s}_{u}(z)$ profile derived for the corresponding layers in Lake Zurich by Strupler et al. (2017), and the ${s}_{u}(z)$ profile for the lacustrine sediments of Lake Lucerne obtained using a linear gradient proposed by Strasser et al. (2011; Fig. 9). For compatibility with the data from lake Zurich, the sediment profile represented by the ${s}_{u}$ values in Fig. 9 consists of the lacustrine sediments in the upper 0–5 m followed by the glaciolacustrine sediments down to 7.5 m depth. To represent our data for Lake Lucerne, we used the raw (Fig. 9a) and fitted (power-law model; Fig. 9b) undrained shear strength profiles.

Our experimental data and power-law models fit well the data of Strupler et al. (2017) and show the same trends for ${s}_{u}$ change with the depth for the lacustrine and glaciolacustrine sediments. Our data provide wider standard deviation bounds due to the larger number of CPTu (152 vs. 8 used in Strupler et al. 2017) and thus, gives better coverage of ${s}_{u}$ spatial variability. Minor differences between the trend of ${s}_{u}$ from Strupler et al. (2017) and our dataset can be explained by the slightly different measurement setup and processing parameters: Strupler et al. (2017) used winch-controlled CPTu deployment, ${N}_{kt}=16$, and log equation for the strain-rate correction, while we used the free-fall CPTu deployment, ${N}_{kt}=15$, and the arcsinh equation. As shown in Sammartini et al. (2021), such differences do not cause strong deviations in the obtained ${s}_{u}$ profiles for the investigated type of sediments. The linear relation proposed by Strasser et al. (2011) clearly shows an overestimation of the undrained shear strength, probably related to the limited number of measured sites (only 3). Another reason for the difference might be related to the measurement approach based on the in situ vane shear testing. As noted by Strupler et al. (2017), the application of the gradients for geotechnical parameters (e.g., ${s}_{u}$) tends to work reliably only in areas with little spatial variations in the sediment thickness and properties, and for relatively thin lithological units.

## Conclusions and outlook

In this work, we collected a dataset of 152 CPTu and 49 sediment cores and samples obtained at different subaqueous slopes in Lake Lucerne. Thus, we provide a dense and lake-wide data set of the sediment properties for the potentially unstable slopes in almost the entire Lake Lucerne, measured in situ and in the laboratory. This allows us to quantify the variations in the sediment properties and significantly improve the previous investigations of slope stability that were based on the sparse geotechnical measurements (Strasser et al. 2011; Hilbe and Anselmetti 2015). Based on the obtained data, we develop generalized empirical models of sediment undrained shear strength evolution with depth below the lake floor and derive the 1D static Factor of Safety at measured locations.

The estimated Factors of Safety (FS) allow us to define three classes of subaqueous slopes in Lake Lucerne in terms of their stability: (1) statically unstable slopes with very soft sediments, no obvious ongoing failures but the possibility of creep and a need for a more detailed geotechnical investigation at specific locations at sites Chrüztrichter, Kastanienbaum, and St. Niklausen; (2) statically stable areas which are close to the unstable state and also require a more comprehensive site-specific investigation (St. Niklausen, Weggis, Kastanienbaum, and partly Muota); (3) statically stable areas (deltaic slopes of Ennetbürgen, Muota, Reuss, and previously failed non-deltaic slopes). In general, the majority of the investigated sites are stable in the present-day condition. For applications of the method in other lakes, we recommend performing a comprehensive core analysis and in situ geotechnical investigations, in particular for the sites belonging to groups (1) and (2).

The potential failure plane at the non-deltaic slopes is usually located within the glaciolacustrine clays, and at the deltaic slopes—in the softest sediment layer. The infinite slope method does not allow the proper incorporation of earthquake-shaking time history and our analysis does not account for seismic or sediment loading which might act on the slope and cause liquefaction or critical strain accumulation. This is especially important for deltaic sediments which partly consist of liquefaction-prone sediments. Thus, as a follow-up to this study, we plan to calibrate the infinite slope equation to introduce earthquake ground motion in the slope stability analysis. Moreover, we plan to perform the dynamic testing of all five described sediment lithologies (with a special focus on the deltaic and glaciolacustrine sediments) to better understand possible loading-induced effects in the sediments. This would allow us to model the sediment behavior under variable loading conditions.

The comparison of derived undrained shear-strength models with the data for Lake Zurich (Strupler et al. 2017) confirmed the applicability of the models to other Swiss lakes with similar sedimentation history as long as similar measurement devices and procedures are used. However, additional measurements in other lakes are needed to confirm these findings. As lakes are rarely surveyed with in situ geotechnical instrumentation (e.g., CPTu), our results might be of interest to the community dealing with subaqueous slope stability analysis and tsunami hazard. Laboratory analysis of the geotechnical index properties of target lithologies allows us to compare the sediments from the lakes of interest with the sediments from Lake Lucerne and to verify the applicability of our ${s}_{u}\left(z\right)$ models to them. Moreover, more site-specific models could be developed by sub-selection of sites available in our dataset. This entire dataset is made public in Shynkarenko et al. (2022).

The presented stability analysis is limited to the 1D case because an impact of the interslice forces that act on the slope and potentially can strengthen it is not straightforward to assess. If they were known, then we could extend the analysis to 2D/3D and get a better understanding of the spatial variability of slope stability. Additionally, the incorporation of interslice forces would verify if the slopes which appear as statically unstable in our analysis truly belong to this category. Last but not least, a 2D or 3D analysis would allow us to define the potentially unstable sediment volumes and thus their potential for tsunami generation that cannot be done based on the 1D estimates. Taking into account possible uncertainty sources in our analysis and its 1D nature, we suggest considering obtained FS as rather a qualitative measure to compare the static stability of different sites to set the priorities for a more in-depth investigation. A probabilistic stability analysis, such as in Strupler et al. (2017), might be useful to define the probability of failure for each measured site.

## Supplementary Information

Below is the link to the electronic supplementary material.

Supplementary file1 (PDF 3392 KB)

## Data Availability

The data underlying this article are gathered in the database attached to Shynkarenko, A., Kremer, K., Stegmann, S., Lontsi, A. M., Roesner, A., Hammerschmidt, S., Kopf, A., Fäh, D. (2022). In situ and laboratory geotechnical investigations (CPTu, sediment coring) performed in Lake Lucerne (Switzerland) in 2018–2020, Report, ETH Zürich, https://doi.org/10.3929/ethz-b-000505160.
