# Supplementary material for: Geotechnical characterization and stability analysis of subaqueous slopes in Lake Lucerne (Switzerland)
Source: Nat Hazards (Dordr). 2022 Mar 29;113(1):475–505. doi: 10.1007/s11069-022-05310-1 (PMC9334397; doi:10.1007/s11069-022-05310-1)
Supplement: Supplementary file 1 — Supplementary file1 (PDF 3392 KB) [file 11069_2022_5310_MOESM1_ESM.pdf]

## Appendix

### Appendix 1. The examples of the undrained shear strength profile and Factor of Safety variation with depth for moraine ridge slope (NA-CPT-02) and deltaic slope (MU-CPT-03).

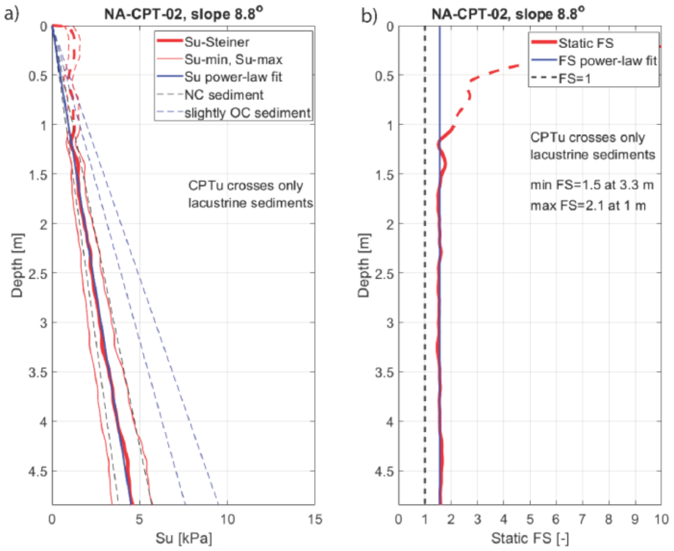


**Fig. 10 a** Undrained shear strength $s_u$ profile for the CPTu NA-CPT-02 (at moraine ridge slope). The red lines show the average $s_u$ profile (thick line, $N_{kt}$=15) and its possible variation for the upper and lower limits of $N_{kt}$ (thin lines, $N_{kt}$ =20 and 12, respectively). The continuous blue line shows the power-law fit of the $s_u$ profile. The upper 1 m of the profile is characterized by the overestimated shear strength and should not be used for any interpretation. The black and blue dashed lines show the $s_u$ ranges for the normally consolidated (NC) and overconsolidated (OC) sediments, respectively. **b** Factor of Safety (FS) for NA-CPT-02 measurement point: the red line shows an estimate based on the average experimental $s_u$ profile, the continuous blue line shows the FS estimated for the power-law fit of the $s_u$ profile. The vertical dashed black line shows the FS=1, which separates the statically stable (FS>1) and unstable (FS≤1) states of the slope

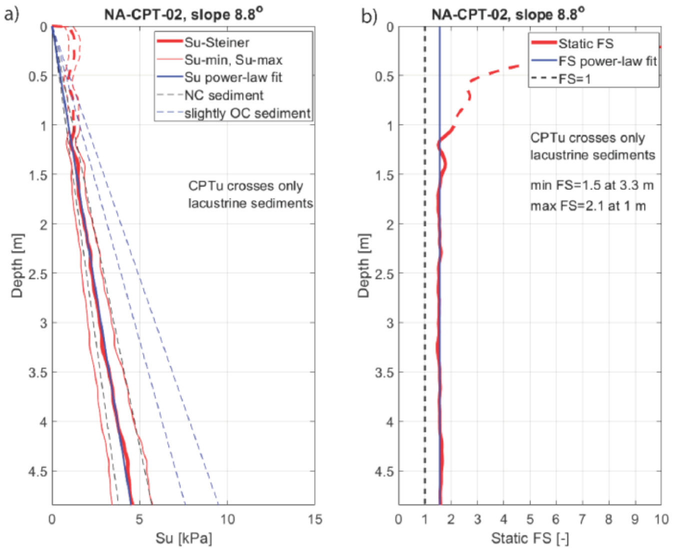


**Fig. 11 a** Undrained shear strength $s_u$ profile for the CPTu MU-CPT-03 (at deltaic slope). The red lines show the average $s_u$ profile (thick line, $N_{kt}$=15) and its possible variation for the upper and lower limits of $N_{kt}$ (thin lines, $N_{kt}$ =20 and 12, respectively). The continuous blue line shows the power-law fit of the $s_u$ profile. The upper 1 m of the profile is characterized by the overestimated shear strength and should not be used for any interpretation. The black and blue dashed lines show the $s_u$ ranges for the normally consolidated (NC) and overconsolidated (OC) sediments, respectively. The dotted blue line shows the boundaries between the sediment units. **b** Factor of Safety (FS) for MU-CPT-03 measurement point: the red line shows an estimate based on the average experimental $s_u$ profile, the continuous blue line shows the FS estimated for the power-law fit of the $s_u$ profile. The vertical dashed black line shows the FS=1, which separates the statically stable (FS>1) and unstable (FS≤1) states of the slope

## Appendix 2. Comparison of the cumulative percentage of CPTu measurements vs. RMSE for the power-law and linear fit of the $s_u(z)$ profiles

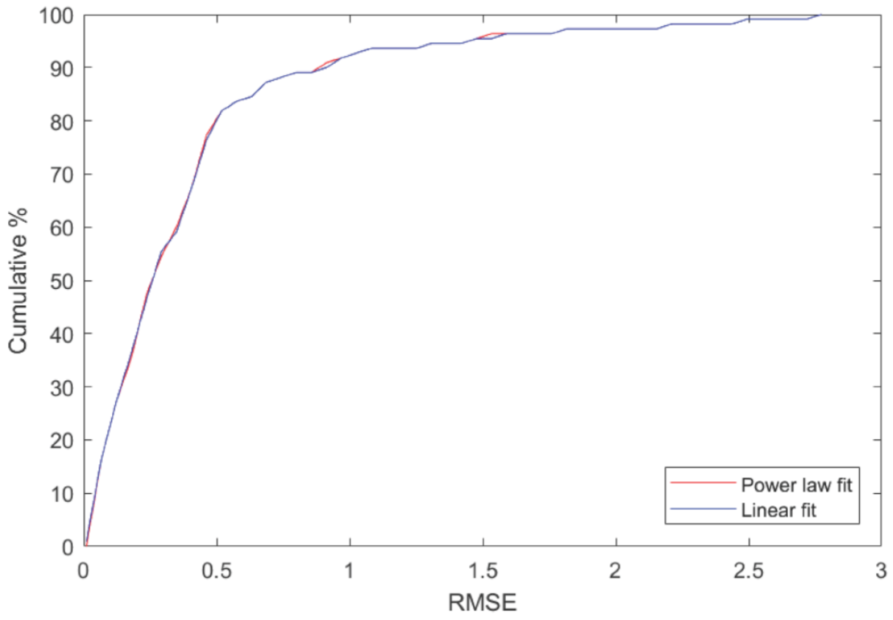


**Fig. 12** Comparison of the cumulative percentage of CPTu measurements which have corresponding RMSE values for the power-law and linear fit of the $s_u(z)$ profiles. Similar performance is observed for both fits (i.e. similar number of the $s_u(z)$ profiles fitted using the power-law and linear relation have an RMSE in the given range)

## Appendix 3. Fitting of the $s_u(z)$ for different lithological units by the power-law relations

Before providing the generalized $s_u(z)$ equations for each lithology, we removed the obvious CPTu-outliers based on their visual inspection. Table 3 shows the corresponding number of CPTu drops.

**Table 3** The number of CPTu drops (with and without outliers) crossing different lithologies

| Lithology | Total # of CPTu | # of CPTu (w/o outliers) | # of outliers | Description of the outliers |
|---|---|---|---|---|
| Lacustrine | 97 | 74 | 23 | 10 CPTu lay in the slope-basin transition zone (possible disturbance); 9 are too short; 4 have erratic spikes |
| Glaciolacustrine | 63 | 52 | 11 | 11 CPTu are too short or have erratic spikes or lay in the slope-basin transition zone |
| Deltaic (background trend) | 35 | 22 | 13 | 13 CPTu are too short or have prevailing spikes which correspond to a stiffer material |

## Comparison of the lacustrine, glaciolacustrine and deltaic sediments: raw data and power-law models

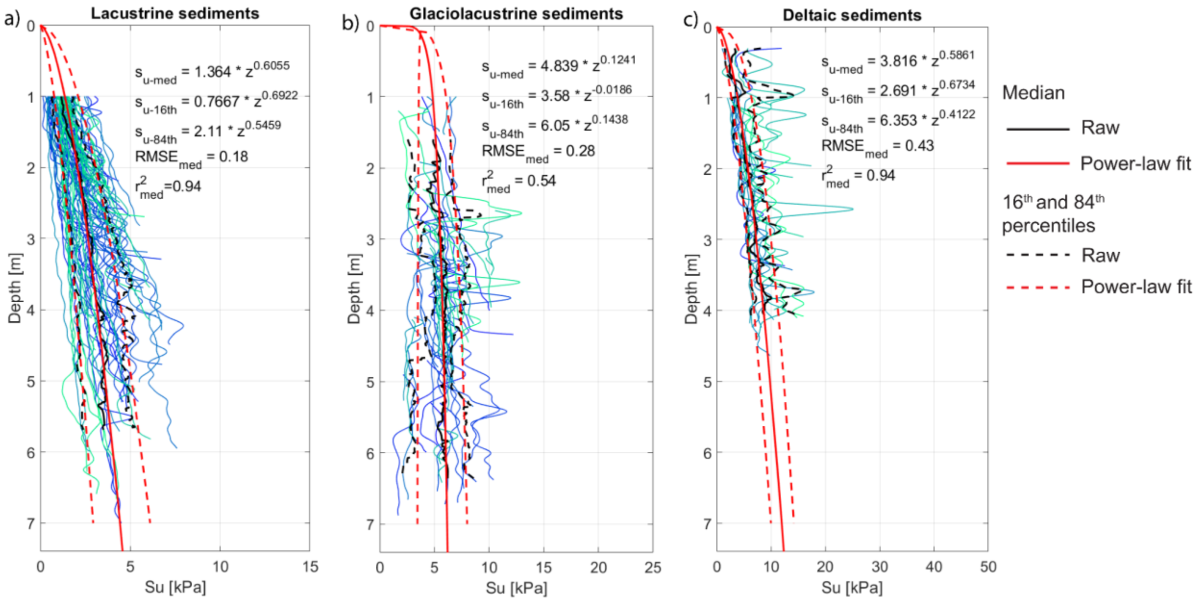


**Fig. 13** Comparison of the median with 16th and 84th percentiles for the **a** lacustrine, **b** glaciolacustrine, and **c** deltaic sediments. A clear difference is observed in the $s_u$ trend for the presented sediment lithotypes. The black continuous line shows the median, black dashed lines - 16th and 84th percentiles for the raw CPTu data. Red lines show the power-law fit of the before-mentioned experimental curves. The equation, RMSE and $r^2$ correspond to the fit of the median $s_u$ curve

## Examples of tested grouping strategies for different lithologies

Below, we present only the examples of the average $s_u$ curves and corresponding standard deviations for the tested sub-groups of CPTu for different lithologies depending on the slope angle and failures of the slope in the past. The behaviour of the median and percentile curves is very similar and thus not shown here. Each of the tested sub-groups consists of at least 11 CPTu.

### Lacustrine sediments

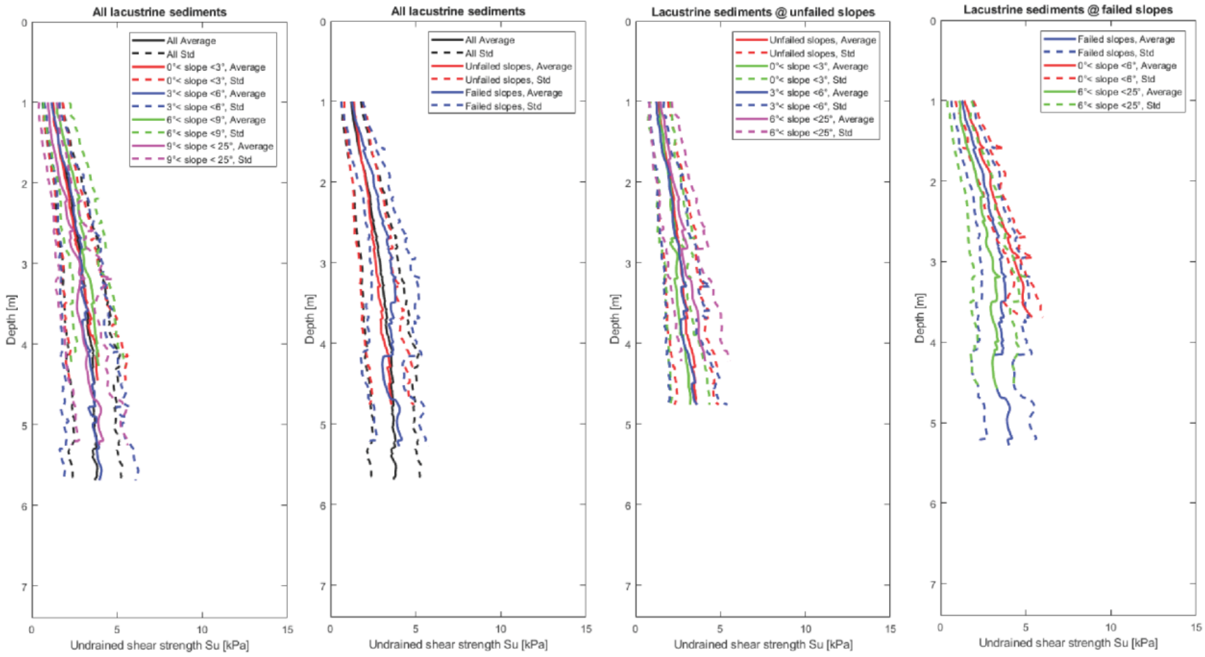


**Fig. 14** Example of overlapped average and standard deviation $s_u$ curves for different groups of CPTu that cross lacustrine sediments (depending on the slope angle and failures in the past)

### Glaciolacustrine sediments

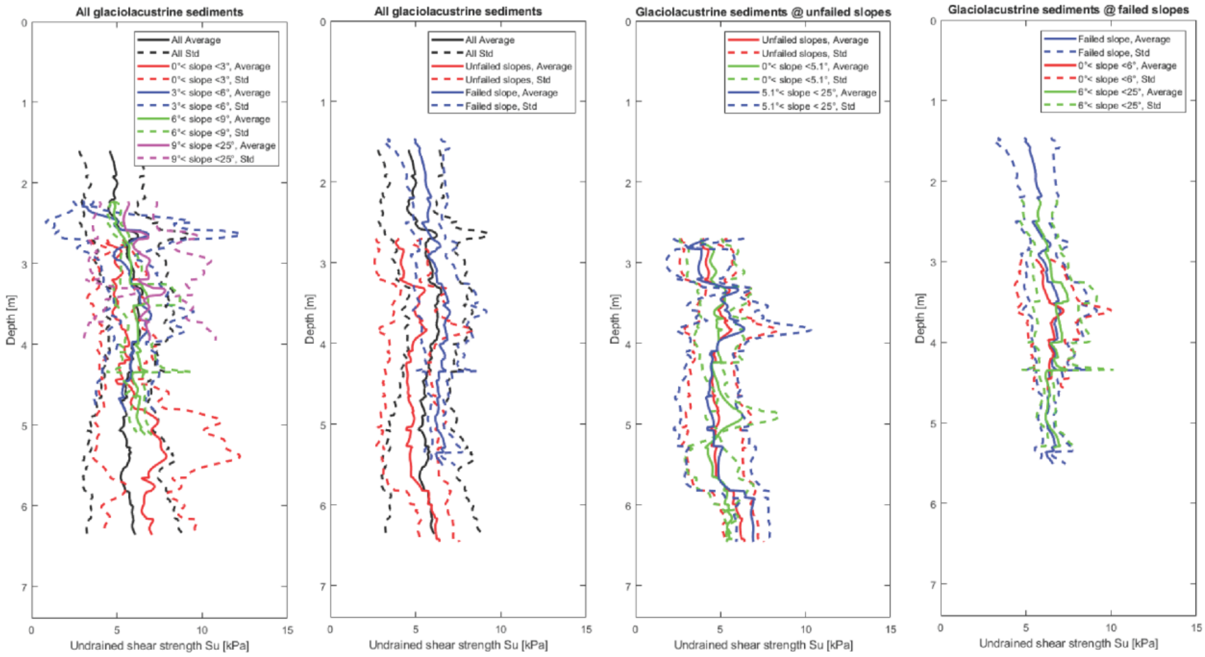


**Fig. 15** Example of overlapped average and standard deviation $s_u$ curves for different groups of CPTu that cross glaciolacustrine sediments (depending on the slope angle and failures in the past)

## Deltaic sediments

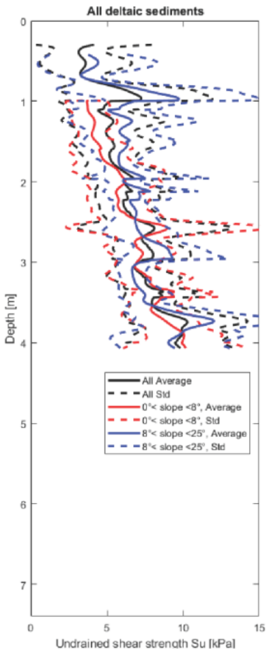


**Fig. 16** An example of overlapped average and standard deviation $s_u$ curves for different groups of CPTu that cross deltaic sediments (depending on the slope angle)

**Table 4** Example of the fitting power-law equations, RMSE and r$^2$ for the average $s_u$ profile for the lacustrine, glaciolacustrine and background deltaic sediments depending on the slope angle and occurrence of the slope failures in the past

| Sediment lithology | State of the slope | Group (slope angle) | $s_{u\,avg}(z) =$ | RMSE | r$^2$ |
|---|---|---|---|---|---|
| Lacustrine | All slopes | >0 | $1.394 \cdot z^{0.6215}$ | 0.17 | 0.96 |
| | | 0-3 | $1.129 \cdot z^{0.8353}$ | 0.05 | 0.996 |
| | | 3-6 | $1.388 \cdot z^{0.621}$ | 0.15 | 0.96 |
| | | 6-9 | $1.525 \cdot z^{0.6698}$ | 0.1 | 0.98 |
| | | 9-25 | $0.9142 \cdot z^{0.8971}$ | 0.15 | 0.97 |
| | Unfailed slopes | >0 | $1.302 \cdot z^{0.6501}$ | 0.05 | 0.99 |
| | | 0-3 | $1.315 \cdot z^{0.5554}$ | 0.11 | 0.96 |
| | | 3-6 | $1.205 \cdot z^{0.6821}$ | 0.11 | 0.97 |
| | | 6-25 | $1.367 \cdot z^{0.7454}$ | 0.08 | 0.99 |
| | Failed slopes | >0 | $1.754 \cdot z^{0.5159}$ | 0.38 | 0.76 |
| | | 0-6 | $1.479 \cdot z^{0.9758}$ | 0.09 | 0.99 |
| | | 6-25 | $1.321 \cdot z^{0.6574}$ | 0.26 | 0.87 |
| Glaciolacustrine | All slopes | >0 | $4.822 \cdot z^{0.1096}$ | 0.46 | 0.12 |
| | | 0-3 | $2.581 \cdot z^{0.5599}$ | 0.55 | 0.69 |
| | | 3-6 | $4.199 \cdot z^{0.2445}$ | 0.81 | 0.18 |
| | | 6-9 | $4.106 \cdot z^{0.2865}$ | 0.30 | 0.67 |
| | | 9-25 | $4.102 \cdot z^{0.4007}$ | 0.41 | 0.54 |
| | Unfailed slopes | >0 | $3.041 \cdot z^{0.322}$ | 0.52 | 0.29 |
| | | 0-5.1 | $3.559 \cdot z^{0.2215}$ | 0.34 | 0.52 |
| | | 5.1-25 | $2.294 \cdot z^{0.5199}$ | 0.86 | 0.28 |
| | Failed slopes | >0 | $5.025 \cdot z^{0.1701}$ | 0.32 | 0.58 |
| | | 0-6 | $4.765 \cdot z^{0.2114}$ | 0.23 | 0.49 |
| | | 6-25 | $5.554 \cdot z^{0.1181}$ | 0.44 | 0.09 |
| Deltaic | All slopes | >0 | $4.449 \cdot z^{0.5158}$ | 0.63 | 0.89 |
| | | 0-8 | $3.29 \cdot z^{0.7612}$ | 0.5 | 0.93 |
| | | 8-25 | $5.251 \cdot z^{0.3691}$ | 0.96 | 0.7 |

## Appendix 4. Results of static stability analysis for each CPTu location (Factor of Safety FS estimated for the raw $s_u(z)$ profile and power-law fit to the $s_u(z)$ profile)

**Table 5** Results of static stability analysis for each CPTu location (Factor of Safety FS for the raw $s_u(z)$ profile and power-law fit to the $s_u(z)$ profile)

| # | CPT name | X [m] | Y [m] | Slope angle [°] | min FS [-] | |
|---|---|---|---|---|---|---|
| | | | | | Estimated for the raw $s_u$ profile | Estimated for the $s_u$ profile fitted with power-law |
| 1 | CHI-CPT-01 | 2684493 | 1204229 | 8.57 | 6.38 | 6.55 |
| 2 | CHI-CPT-02 | 2684515 | 1204123 | 19.75 | 3.06 | 3.14 |
| 3 | CHR-CPT-02 | 2672279 | 1208972 | 3.21 | 3.46 | 3.9 |
| 4 | CHR-CPT-10 | 2672140 | 1208701 | 1.34 | 10.03 | 13.67 |
| 5 | CHR-CPT-12 | 2671508 | 1208253 | 0.91 | 8.27 | 12.43 |
| 6 | CHR-CPT-13 | 2671735 | 1208313 | 11.27 | 2.53 | 2.33 |
| 7 | CHR-CPT-14 | 2671802 | 1208356 | 7.23 | 3.06 | 3.43 |
| 8 | CHR-CPT-15 | 2671791 | 1208439 | 25.71 | 56.64 | 999 |
| 9 | CHR-CPT-16 | 2671900 | 1208641 | 3.49 | 1.42 | 2.7 |
| 10 | CHR-CPT-17 | 2671833 | 1208503 | 9.53 | 0.96 | 1.18 |
| 11 | CHR-CPT-18 | 2671799 | 1208632 | 10.77 | 0.85 | 1.34 |
| 12 | CHR-CPT-19 | 2671714 | 1208621 | 26.71 | 1.67 | 1.77 |
| 13 | CHR-CPT-20 | 2671851 | 1208965 | 19.12 | 0.69 | 0.72 |
| 14 | CHR-CPT-21 | 2671747 | 1208987 | 5.78 | 0.51 | 0.76 |
| 15 | CHR-CPT-22 | 2671855 | 1209193 | 1.83 | 2.32 | 6.46 |
| 16 | CHR-CPT-23 | 2671684 | 1209255 | 2.37 | 3.3 | 3.75 |
| 17 | CHR-CPT-24 | 2671893 | 1209288 | 2.37 | 3.67 | 3.94 |
| 18 | EN-CPT-01 | 2674741 | 1204056 | 10.49 | 0.81 | 1.5 |
| 19 | EN-CPT-02 | 2674869 | 1204041 | 7.5 | 2.43 | 2.92 |
| 20 | EN-CPT-03 | 2674998 | 1204023 | 15.45 | 2.54 | 3.24 |
| 21 | EN-CPT-04 | 2675121 | 1204001 | 3.03 | 4.06 | 4.61 |
| 22 | EN-CPT-05 | 2675308 | 1204015 | 0.98 | 283.35 | 601.95 |
| 23 | EN-CPT-06 | 2675447 | 1204017 | 6.56 | 11.4 | 29.59 |
| 24 | EN-CPT-08 | 2675767 | 1204032 | 3.66 | 9.84 | 25.13 |
| 25 | EN-CPT-12 | 2676410 | 1204142 | 0.33 | 413.44 | 919.78 |
| 26 | EN-CPT-13 | 2676573 | 1204182 | 3.96 | 22.6 | 22.78 |
| 27 | EN-CPT-14 | 2676763 | 1204193 | 4.27 | 6.5 | 20.91 |
| 28 | EN-CPT-15 | 2676975 | 1204239 | 1.37 | 42.49 | 44.24 |
| 29 | EN-CPT-17 | 2675366 | 1204617 | 0.6 | 32.92 | 36.9 |
| 30 | EN-CPT-18 | 2675525 | 1204498 | 3.91 | 4.05 | 4.38 |
| 31 | EN-CPT-19 | 2675639 | 1204415 | 4.47 | 3.57 | 3.75 |

| # | CPT name | X [m] | Y [m] | Slope angle [°] | min FS [-] | |
|---|---|---|---|---|---|---|
| | | | | | Estimated for the raw $s_u$ profile | Estimated for the $s_u$ profile fitted with power-law |
| 32 | EN-CPT-20 | 2674771 | 1204303 | 6.64 | 2.79 | 2.23 |
| 33 | EN-CPT-21 | 2675082 | 1204163 | 3.35 | 4.67 | 4.75 |
| 34 | EN-CPT-22 | 2675246 | 1204085 | 1.98 | 8.43 | 8.78 |
| 35 | KB-CPT-03b-ff | 2668367 | 1205023 | 5.02 | 3.08 | 3.13 |
| 36 | KB-CPT-03b-wc | 2668351 | 1205026 | 6.02 | 4.03 | 4.6 |
| 37 | KB-CPT-10 | 2668128 | 1205338 | 2.97 | 2.63 | 3.36 |
| 38 | KB-CPT-11 | 2668214 | 1205236 | 2.01 | 2.21 | 3.45 |
| 39 | KB-CPT-12 | 2668365 | 1205141 | 0.59 | 12.45 | 17.86 |
| 40 | KB-CPT-13 | 2668460 | 1205049 | 5.79 | 1.97 | 2.56 |
| 41 | KB-CPT-14 | 2668558 | 1205021 | 6.94 | 1.92 | 1.96 |
| 42 | KB-CPT-15 | 2668671 | 1204947 | 7.25 | 0.2 | 1.3 |
| 43 | KB-CPT-16 | 2668790 | 1204932 | 7.95 | 1.82 | 1.93 |
| 44 | KB-CPT-17 | 2668706 | 1204829 | 17.06 | 0.77 | 0.92 |
| 45 | KB-CPT-18 | 2668754 | 1204885 | 11.9 | 0.27 | 1.07 |
| 46 | KB-CPT-19 | 2669069 | 1204838 | 0.76 | 7.05 | 15.24 |
| 47 | KB-CPT-30 | 2668210 | 1204624 | 0.4 | 33.65 | 52.71 |
| 48 | KB-CPT-31 | 2668215 | 1204683 | 3.02 | 5.03 | 6.01 |
| 49 | KB-CPT-32 | 2668218 | 1204741 | 7.62 | 1.26 | 1.98 |
| 50 | KB-CPT-33 | 2668219 | 1204861 | 3.25 | 1.69 | 4.54 |
| 51 | KB-CPT-34 | 2668236 | 1204971 | 5.26 | 2.84 | 2.97 |
| 52 | KB-CPT-35 | 2668315 | 1204998 | 5.31 | 2.09 | 2.39 |
| 53 | KB-CPT-36ff | 2668222 | 1205119 | 0.73 | 13.25 | 14.49 |
| 54 | KB-CPT-36wc | 2668220 | 1205120 | 0.73 | 17.28 | 17.38 |
| 55 | KE-CPT-02 | 2670299 | 1205723 | 20.88 | 1.36 | 1.25 |
| 56 | KE-CPT-03 | 2670202 | 1205724 | 27.74 | 29.8 | 999 |
| 57 | KE-CPT-06 | 2670628 | 1206452 | 20.38 | 0.56 | 0.92 |
| 58 | KE-CPT-07 | 2670119 | 1205793 | 7.14 | 4.94 | 4.98 |
| 59 | KE-CPT-08 | 2669973 | 1205788 | 4.62 | 7.35 | 7.59 |
| 60 | KE-CPT-09 | 2669817 | 1205838 | 5.16 | 1.25 | 3.7 |
| 61 | KE-CPT-10 | 2670581 | 1206579 | 6.3 | 2.62 | 2.83 |
| 62 | KE-CPT-11 | 2670301 | 1205637 | 6.03 | 2.65 | 2.82 |
| 63 | KE-CPT-12(07-001) | 2670621 | 1206803 | 2.52 | 4.88 | 8.25 |
| 64 | MU-CPT-01 | 2687502 | 1205619 | 14.12 | 1.55 | 1.83 |
| 65 | MU-CPT-02 | 2687500 | 1205538 | 7.31 | 1.96 | 2.43 |
| 66 | MU-CPT-03 | 2687476 | 1205472 | 8.64 | 1.86 | 2.11 |

| # | CPT name | X [m] | Y [m] | Slope angle [°] | min FS [-] | |
|---|---|---|---|---|---|---|
| | | | | | Estimated for the raw $s_u$ profile | Estimated for the $s_u$ profile fitted with power-law |
| 67 | MU-CPT-04 | 2687437 | 1205406 | 12.18 | 1.69 | 1.7 |
| 68 | MU-CPT-05 | 2687403 | 1205323 | 9.31 | 1.42 | 1.64 |
| 69 | MU-CPT-06 | 2687364 | 1205245 | 2.67 | 5.17 | 5.49 |
| 70 | MU-CPT-07 | 2687304 | 1205180 | 5.37 | 2.99 | 3.23 |
| 71 | MU-CPT-08 | 2687199 | 1204948 | 2.01 | 13.31 | 14.15 |
| 72 | MU-CPT-09 | 2686959 | 1204877 | 1.41 | 16.91 | 18 |
| 73 | MU-CPT-10 | 2686462 | 1204753 | 0.83 | 17.48 | 24.38 |
| 74 | MU-CPT-11 | 2686530 | 1204651 | 1.13 | 21 | 29.36 |
| 75 | MU-CPT-12 | 2687976 | 1205221 | 10.92 | 2.81 | 4.48 |
| 76 | MU-CPT-13 | 2688038 | 1205095 | 9.08 | 1.59 | 5.6 |
| 77 | MU-CPT-14 | 2688002 | 1204990 | 11.89 | 2.63 | 2.91 |
| 78 | NA-CPT-01 | 2678556 | 1206274 | 6.23 | 3.71 | 3.75 |
| 79 | NA-CPT-02 | 2678096 | 1205989 | 8.82 | 1.48 | 1.57 |
| 80 | NI-CPT-01 | 2668439 | 1208745 | 8.75 | 1.09 | 1.46 |
| 81 | NI-CPT-02 | 2668518 | 1208773 | 18.43 | 0.26 | 0.47 |
| 82 | NI-CPT-05 | 2668824 | 1208862 | 1.38 | 4.48 | 6.27 |
| 83 | NI-CPT-06 | 2668951 | 1208879 | 0.48 | 7.48 | 18.55 |
| 84 | NI-CPT-07 | 2668492 | 1208972 | 13.23 | 0.35 | 0.5 |
| 85 | NI-CPT-08 | 2668547 | 1208931 | 11.35 | 0.32 | 0.5 |
| 86 | NI-CPT-09 | 2668626 | 1208916 | 10.91 | 0.43 | 1.29 |
| 87 | NI-CPT-10 | 2668672 | 1208944 | 16.85 | 2.15 | 1.73 |
| 88 | NI-CPT-11 | 2668705 | 1208943 | 16.1 | 8.96 | 16.26 |
| 89 | NI-CPT-12 | 2668447 | 1208606 | 6.5 | 1.06 | 1.8 |
| 90 | NI-CPT-13 | 2668632 | 1208469 | 5.25 | 1.46 | 2.41 |
| 91 | NI-CPT-14 | 2668516 | 1208415 | 5.72 | 8.62 | 6.31 |
| 92 | RE-CPT-01 | 2688330 | 1194326 | 1.92 | 10.74 | 12.91 |
| 93 | RE-CPT-01a | 2688335 | 1194301 | 11.4 | 2.18 | 3.17 |
| 94 | RE-CPT-02 | 2688364 | 1194427 | 0.43 | 50.98 | 60.1 |
| 95 | RE-CPT-03 | 2688374 | 1194566 | 1.31 | 16.4 | 32.05 |
| 96 | RE-CPT-05 | 2690044 | 1195978 | 22.45 | 1.34 | 1.23 |
| 97 | RE-CPT-06a | 2689978 | 1195922 | 18.15 | 1.93 | 2.21 |
| 98 | RE-CPT-06b | 2689965 | 1195917 | 18.58 | 0.9 | 1.42 |
| 99 | RE-CPT-07 | 2688509 | 1195576 | 0.86 | 28.68 | 31.64 |
| 100 | WE-CPT-02 | 2673895 | 1208514 | 8.77 | 0.98 | 1.1 |
| 101 | WE-CPT-03 | 2673909 | 1208853 | 4.58 | 2.56 | 2.59 |

| # | CPT name | X [m] | Y [m] | Slope angle [°] | min FS [-] | |
|---|---|---|---|---|---|---|
| | | | | | Estimated for the raw $s_u$ profile | Estimated for the $s_u$ profile fitted with power-law |
| 102 | WE-CPT-03-3 | 2673896 | 1208866 | 6.58 | 2.29 | 4.35 |
| 103 | WE-CPT-04 | 2674409 | 1208839 | 5.39 | 1.06 | 1.31 |
| 104 | WE-CPT-05 | 2674466 | 1208686 | 13.47 | 2.45 | 2.64 |
| 105 | WE-CPT-06(09) | 2674499 | 1208448 | 6.2 | 1.63 | 1.63 |
| 106 | WE-CPT-06(13) | 2674512 | 1208486 | 4.02 | 1.45 | 1.34 |
| 107 | WE-CPT-06a | 2674491 | 1208491 | 1.67 | 6.39 | 6.68 |
| 108 | WE-CPT-07ff | 2674562 | 1208249 | 10.1 | 2.42 | 2.46 |
| 109 | WE-CPT-07wc | 2674539 | 1208240 | 8.09 | 10.56 | 10.87 |
| 110 | WE-CPT-08 | 2674176 | 1208432 | 6.45 | 5.42 | 6.54 |
| 111 | WE-CPT-08a | 2674202 | 1208446 | 6.18 | 2.65 | 2.94 |
| 112 | WE-CPT-08b | 2674186 | 1208433 | 5.86 | 2.6 | 2.99 |
| 113 | WE-CPT-08c | 2674199 | 1208431 | 5.11 | 3.41 | 3.72 |
| 114 | WE-CPT-08d | 2674180 | 1208437 | 5.52 | 1.74 | 2.73 |
| 115 | WE-CPT-08e | 2674166 | 1208437 | 6.13 | 1.76 | 2.53 |
| 116 | WE-CPT-09 | 2674837 | 1208887 | 5.64 | 1.12 | 1.16 |
| 117 | WE-CPT-10 | 2674858 | 1208796 | 16.01 | 40.01 | N/A |
| 118 | WE-CPT-11 | 2674960 | 1208618 | 2.31 | 5.59 | 5.48 |
| 119 | WE-CPT-11a | 2675049 | 1208395 | 1.77 | 6.19 | 6.09 |
| 120 | WE-CPT-11b | 2674948 | 1208651 | 0.78 | 16.43 | 16.39 |
| 121 | WE-CPT-12 | 2675094 | 1208334 | 8.09 | 5.17 | 5.27 |
| 122 | WE-CPT-13 | 2675132 | 1208297 | 9.81 | 4.28 | 4.28 |
| 123 | WE-CPT-14 | 2674100 | 1208439 | 4.88 | 2.15 | 3.68 |
| 124 | WE-CPT-15 | 2674357 | 1208444 | 3.41 | 7.44 | 9.96 |
| 125 | WE-CPT-16 | 2674628 | 1208462 | 1.73 | 9.97 | 11.45 |
| 126 | WE-CPT-17 | 2674889 | 1208466 | 2.29 | 10.25 | 10.23 |
| 127 | WE-CPT-20 | 2675167 | 1208530 | 1.13 | 13.38 | 16.97 |
| 128 | WE-CPT-21 | 2674853 | 1208510 | 1.19 | 12.44 | 15.77 |
| 129 | WE-CPT-22 | 2674841 | 1208562 | 3.82 | 3.44 | 5.8 |
| 130 | WE-CPT-23 | 2674805 | 1208569 | 8.8 | 1.95 | 2.15 |
| 131 | WE-CPT-24 | 2674769 | 1208647 | 3.22 | 3.61 | 7.19 |
| 132 | WE-CPT-25 | 2675002 | 1208497 | 3.32 | 1.69 | 1.91 |
| 133 | WE-CPT-26 | 2673926 | 1208434 | 2.16 | 3.99 | 4.02 |
| 134 | WE-CPT-27 | 2675072 | 1208500 | 2.99 | 1.17 | 1.63 |
| 135 | WE-CPT-28 | 2674765 | 1208635 | 3.01 | 4.98 | 6.56 |
| 136 | WE-CPT-29 | 2674792 | 1208604 | 5.47 | 3.4 | 4.42 |

| # | CPT name | X [m] | Y [m] | Slope angle [°] | min FS [-] | |
|---|---|---|---|---|---|---|
| | | | | | Estimated for the raw $s_u$ profile | Estimated for the $s_u$ profile fitted with power-law |
| 137 | WE-obs1 | 2673351 | 1208642 | 11.04 | 1.3 | 1.17 |
| 138 | WE-obs2 | 2673691 | 1208658 | 4.64 | 0.93 | 1.85 |
| 139 | WE-obs3 | 2673922 | 1208977 | 4.91 | 2.38 | 2.53 |
| 140 | WE-obs4 | 2673949 | 1208314 | 3.06 | 2.44 | 2.67 |

**Appendix 5. Graphical results of static stability analysis for each CPTu location (zoom to each site)**

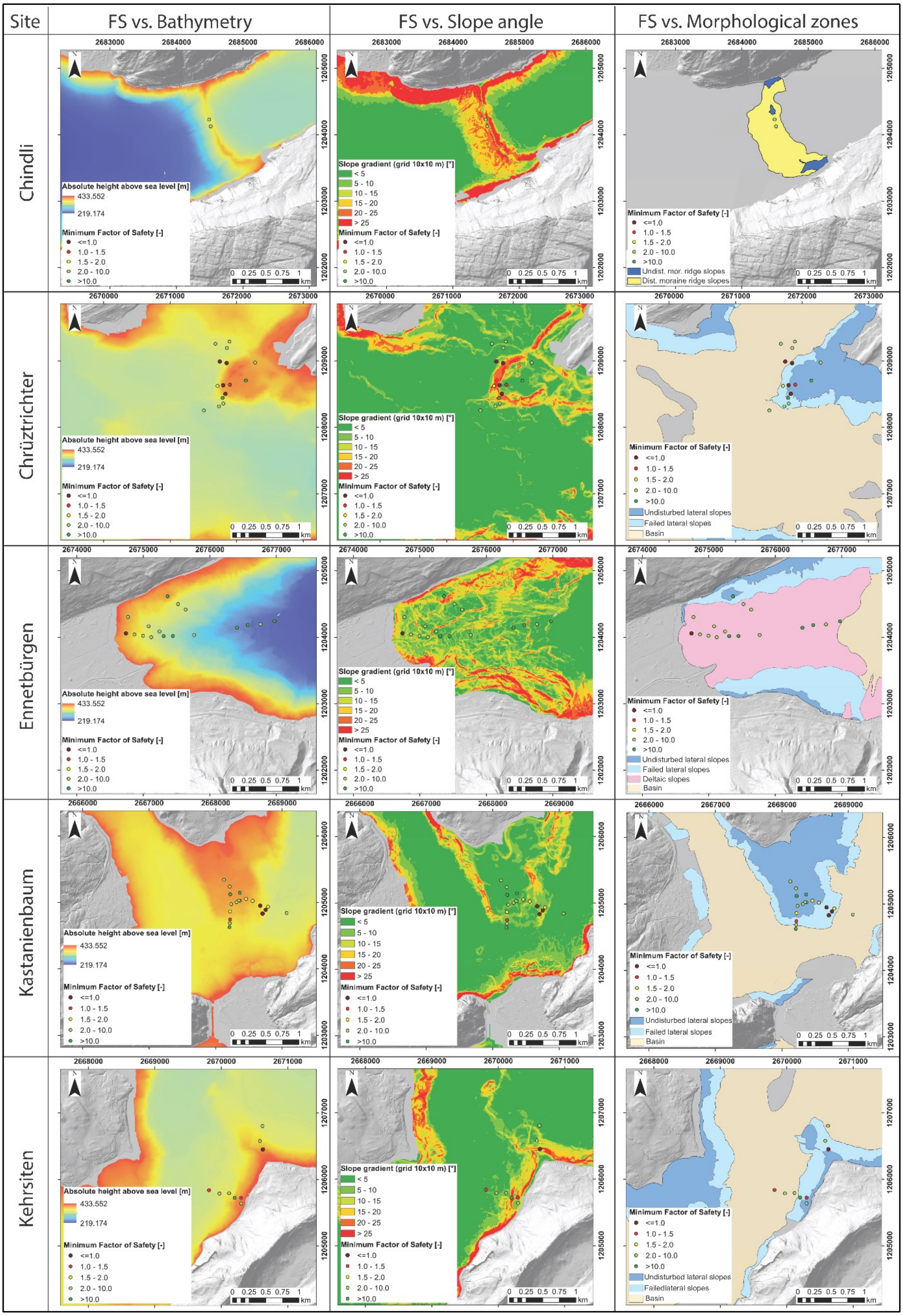

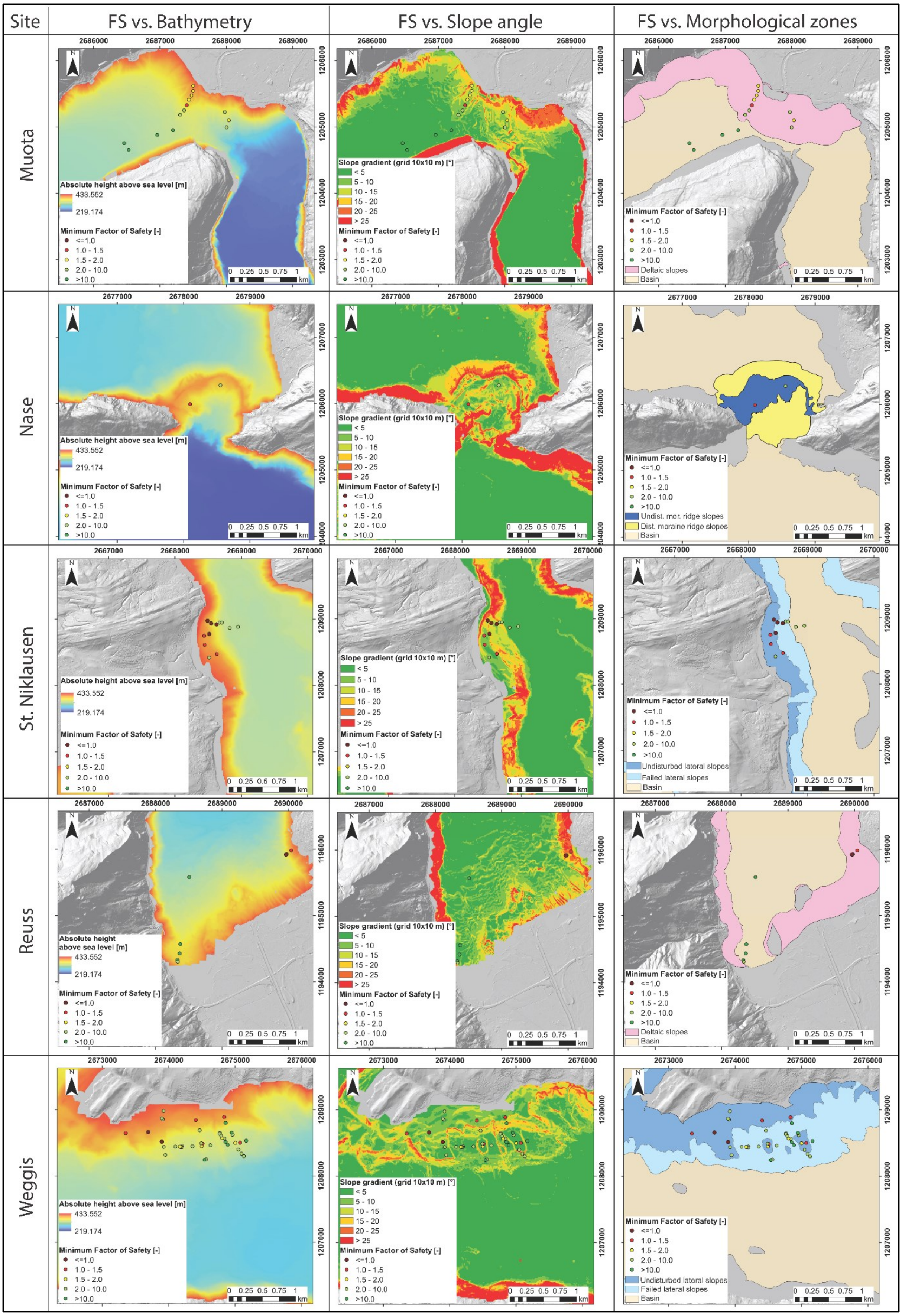
Site
FS vs. Bathymetry
FS vs. Slope angle
FS vs. Morphological zones
Muota
Nase
St. Niklausen
Reuss
Weggis
Absolute height above sea level [m]
433.552
219.174
Minimum Factor of Safety [-]
<=1.0
1.0 - 1.5
1.5 - 2.0
2.0 - 10.0
>10.0
Slope gradient (grid 10x10 m) [°]
< 5
5 - 10
10 - 15
15 - 20
20 - 25
> 25
Deltaic slopes
Basin
Undist. mor. ridge slopes
Dist. moraine ridge slopes
Undisturbed lateral slopes
Failed lateral slopes
0 0.25 0.5 0.75 1
km
N
